# Mitochondrial Uncoupling: A Key Controller of Biological Processes in Physiology and Diseases

**DOI:** 10.3390/cells8080795

**Published:** 2019-07-30

**Authors:** Stéphane Demine, Patricia Renard, Thierry Arnould

**Affiliations:** 1 ULB Center for Diabetes Research, University of Brussels (ULB), 1070 Brussels, Belgium; 2 Laboratory of Biochemistry and Cell Biology (URBC), NARILIS (Namur Research Institute for Life Sciences), University of Namur (UNamur), 5000 Namur, Belgium

**Keywords:** mitochondrial uncoupling, uncoupler, cell signaling, cell death, apoptosis, autophagy, protein secretion, oxidative stress, adipocyte browning, physical exercise

## Abstract

Mitochondrial uncoupling can be defined as a dissociation between mitochondrial membrane potential generation and its use for mitochondria-dependent ATP synthesis. Although this process was originally considered a mitochondrial dysfunction, the identification of UCP-1 as an endogenous physiological uncoupling protein suggests that the process could be involved in many other biological processes. In this review, we first compare the mitochondrial uncoupling agents available in term of mechanistic and non-specific effects. Proteins regulating mitochondrial uncoupling, as well as chemical compounds with uncoupling properties are discussed. Second, we summarize the most recent findings linking mitochondrial uncoupling and other cellular or biological processes, such as bulk and specific autophagy, reactive oxygen species production, protein secretion, cell death, physical exercise, metabolic adaptations in adipose tissue, and cell signaling. Finally, we show how mitochondrial uncoupling could be used to treat several human diseases, such as obesity, cardiovascular diseases, or neurological disorders.

## 1. Introduction

According to the chemiosmotic theory developed by Peter Mitchell, mitochondrial electron transfer is accompanied by proton fluxes and coupled through the redox proton pumps mediated by mitochondrial complexes (CI, CIII, and CIV) [1]. Mitochondrial electron transfer is usually assessed by oxygen consumption measurement and establishes electrochemical potential that is finally used by F_0_-F_1_ ATP synthase to generate ATP [2]. However, not all potential energy is transformed/converted into ATP by the mitochondrial rotor, as some degree of uncoupling relative to the substrate-dependent coupling stoichiometry is observed during electrochemical energy transformation into ATP. This incomplete coupling can be explained by several processes: basal and inducible proton leak, electron leak, and electron slip. Protons can directly escape from the inner mitochondrial space by diffusion through the inner mitochondrial membrane, a process named basal proton leak. This process essentially depends on the composition of the inner mitochondrial membrane and could account for up to 30–50% of the resting cellular metabolic rate [3]. Second, this process can also be regulated/induced by a dedicated set of proteins: UCPs (Uncoupling Proteins) or ANTs (Adenine Nucleotide Translocases). The mechanisms of these proteins will be covered later in this review. Although under normal circumstances, electrons are ultimately transferred to an oxygen to form water, electrons can also leak from the electron transfer chain and lead to the production of superoxide anion (O_2_^•−^) or hydroperoxyl radical (HO_2_^•^) [3]. Electron slip consists of the transfer of electrons through the respiratory complexes without proton pumping [3,4]. Interested readers will find more details about the differences between proton leak, electron slip, and uncoupling in previous reports [5].

Mitochondrial uncoupling can be caused by a variety of conditions and molecules that exert an influence not only on proton leak and cation cycling but also on proton slip within the proton pumps and on the structural integrity of the mitochondria. In addition, the use of protonophores represents an experimental uncoupling intervention to assess the transition from a well-coupled to a noncoupled state of mitochondrial respiration [6]. These authors have clearly defined what should be understood by coupling and uncoupling (or dyscoupling) mitochondrial respiration [6]. In this review, we will cover recent advances in the understanding of the effects of natural and artificial mitochondrial uncoupling in the regulation of biological processes and diseases such as obesity, cardiovascular diseases, or neurological disorders.

## 2. Mitochondrial Uncouplers

### 2.1. UCPs and ANTs

UCPs are a protein family composed of 5 members (UCP-1–UCP-5) in humans. UCP-1 is a transmembrane protein localized in the inner mitochondrial membrane catalyzing the transport of protons across the mitochondrial membrane and thereby inducing mitochondrial uncoupling [7]. UCP-1 function is tightly regulated. First, purine nucleotides, such as GDP, directly bind UCP-1 and lead to its inactivation [8]. Second, free fatty acids (FFAs) are well-known to promote UCP-1-dependent protein leak [8,9]. In addition, UCP-1 activity is also tightly modulated by phosphorylation, a phenomenon shown to increase in response to cold in rats [10]. However, to date, the precise molecular mechanism involved in UCP-1 mediated protein leak is not yet clear. Four different models have been proposed for UCP-1-dependent proton transport [11]. In the first model (competition model), FFAs compete with purine nucleotides to bind to UCP-1. Once bound, FFAs induce proton transport through the channel by an as of yet unclear mechanism [11]. In the second model, FFAs do not compete with purine nucleotides but are directly used by UCP-1 as a co-factor to complete a proton transfer pathway through the protein [11]. In the third model, the cycling model, FFAs are directly imported by UCP-1 in the inner mitochondrial space. Once protonated, FFAs can flip back to the mitochondrial matrix by crossing the mitochondrial inner membrane in a UCP-1-independent mechanism [11]. Finally, in the fourth model, namely the shuttling model, both protonated and deprotonated FFAs are directly transported by UCP-1. However, long chain FFAs cannot cross entirely through UCP-1 due do their hydrophobicity but can still “capture” protons and thus induce net proton leak through UCP-1 [11]. These models have been deeply discussed in a dedicated review [11].

Importantly, although strong evidence of the mitochondrial uncoupling property is clear for UCP-1 [7,12], it is still heavily debated for the other UCPs [13,14,15]. Regarding UCP-2, Bouillaud and collaborators first suggested that this protein could have uncoupling properties in yeast expression systems [16], but they later reported that it does not possess any uncoupling effect in mice [17]. However, others found that UCP-2 could indeed induce mitochondrial uncoupling by essentially acting as a fatty acid flippase transporter [18]. The same authors suggested that UCP-2 activity could be completely blocked by GDP at the basal state (100 µM, which corresponds to the basal intracellular GDP/GTP concentration) [18]. UCP-2 could then only be activated under some circumstances, such as a massive entry of FFAs into mitochondria [18]. Regarding UCP-3, several groups reported that UCP-3 has no clear uncoupling effect as it does, for instance, in CHO cells overexpressing this protein [19] or in rat skeletal muscles [20]. However, as for UCP-2, UCP-3 uncoupling activity could only be activated under particular circumstances, such as high availability in FFAs. Importantly, one must keep in mind the endogenous level of expression of each UCP. For instance, UCP-3 expression is expressed at a much lower level than UCP-1 (200–700 fold) in mouse skeletal muscle [21]. Therefore, its function (as well as any other UCP) should be studied in models with similar expression levels. The mitochondrial uncoupling effect observed for these UCPs in an overexpression system could otherwise be likely artefactual. Finally, other functions have been suggested for UCP-2 and UCP-3 and include redox regulation or glucose sensing [13,22].

The second family of uncoupling proteins, namely ANT, comprises 4 members in humans (ANT-1–ANT-4) that catalyze the ATP/ADP exchange across the mitochondrial membrane and play a key role in ATP export to cytosol. Besides this activity, ANT also possesses some uncoupling properties. The molecular mechanism is not entirely clear but has been suggested to depend on the transport of fatty acids [23,24]. Underlining their importance, ANTs can account for up to 50% of basal mitochondrial membrane proton conductance [25]. The structure and function of UCPs and ANTs have been reviewed elsewhere [26,27].

Although synthetic chemicals are often used to induce mitochondrial uncoupling in in vitro studies, biological compounds can also have such properties. Their uncoupling effect could be either direct (by disrupting the mitochondrial proton gradient) or indirect (for instance, by stimulation/regulation of the activity/expression of uncoupling proteins or by altering metabolism and mitochondrial function). Mitochondrial uncouplers can be further divided into two categories: protonophore (an ionophore molecule able to translocate protons) and non-protonophore uncouplers. In the next part of this review, we will discuss the different existing natural and synthetic uncouplers and present their main characteristics. When available, details regarding the mechanisms of action and targets are provided. To help the reader, information relative to mitochondrial uncouplers and their characteristics has been summarized in Table 1.

### 2.2. Natural Uncouplers

FFAs form one of the major class of endogenous mitochondrial uncouplers. They can act through various mechanisms. First, they stimulate directly mitochondrial respiration, as seen in intact isolated brown adipocytes or mitochondria isolated from these cells [28,29]. The FFA protonophoric effect depends on the chain length [30]. FFAs with a carbon chain between C12 and C16 as well as long unsaturated FFAs (length above one-half of the mitochondrial membrane thickness, ±3.5 nm) seem to have the most potent effect [30]. Second, and as discussed before, FFAs have a direct effect on UCP-1 activity. Experiments performed on liposomes enriched in UCP-1 showed that FFAs are a required co-factor for UCP-1-catalyzed proton transport [29], suggesting that the uncoupling effect of FFAs could depend on this channel. It is now largely admitted that in the presence of certain types of FFAs, UCP-1 catalyzes the electrophoretic transport of protons but also performs the electrophoresis of selective anions (reviewed recently [31]). Other studies proved that FFAs, such as palmitate, can physically interact with UCP-1, leading to a change in the protein conformation and inducing *in fine* mitochondrial uncoupling [32]. This study evidences that FFAs would bind and regulate UCP-1 in a competitive manner with nucleotides. However, other experiments challenged these findings, suggesting instead that FFAs can directly act as mitochondrial uncouplers even in the absence of UCP-1 [33,34], although conflicting results were also found [35]. The use of FFAs to stimulate mitochondrial uncoupling could be problematic, as they can also be used as a source of energy. To cope with this problem, the perfluorinated fatty acids perfluorooctane sulfonate and perfluorooctanoate, two metabolically inactive FFAs, were identified as also capable of stimulating the UCP-1 uncoupling function, at least in isolated mouse brown-fat mitochondria [36], but they cannot be metabolized. These FFAs could thus be useful to induce UCP-1 activation, with no or little impact on metabolism. 

UCP activity and/or expression can also be modified by using specific experimental/physiological conditions or compounds. Therefore, the use of these conditions or molecules can induce mitochondrial uncoupling. The transcriptional regulation and activity regulation of UCP-1 has been extensively reviewed recently and will not be discussed in detail here [37]. The most well-known condition to induce *Ucp1* expression in both humans and rodents is cold, essentially by activating β-adrenergic receptors and the cAMP-dependent protein kinase (PKA)-dependent signaling pathway [38,39]. In C57BL/6J mice, cold exposure also upregulates the expression of cAMP responsive element binding protein (CREB)-regulated transcription coactivator 3 (CRTC3) and promotes its nuclear translocation [38]. Treatment with forskolin, an adenylate cyclase activator and thereby an indirect PKA activator, mimics this effect in vitro in isolated mouse brown adipocytes [38]. A second well-known condition to induce UCP-1 activity is by exposition to catecholamines (such as noradrenaline) and subsequent activation of β3 adrenergic receptors. These molecules are also known to upregulate UCP-1 expression in many models, including mouse [40,41], rat [42,43], and human [44] brown adipose tissues.

Capsaicins and their derivatives are red-pepper components and are long known for their capacity to induce the upregulation of uncoupling proteins, even in vivo [62]. The molecular mechanisms are still unclear but could involve sympathetic stimulation [63] or binding to the receptor Trpv1 (transient receptor potential cation channel subfamily V member 1) at the brown adipocyte surface [64]. However, supraphysiologic concentration in capsaicin (100 μM) can also lead to calcium efflux from the ER (Endoplasmic Reticulum) and finally to UPR (Unfolded Protein Response; assessed by increased splicing of XBP1 (X-box binding protein 1) and CHOP (Protein Homologous Protein) expression) [65]. Interestingly, XBP1 expression was found to be positively associated with brown adipogenesis, at least in vitro, in mouse primary brown adipocytes [65]. Of note, capsaicin is also a potent neurotoxin, a high concentration of which could lead to sensory denervation, which impairs the brown adipose tissue (BAT) function [66]. PUFAs such as eicosapentaenoic acid (EPA) and docosahexaenoic acid (DHA) also stimulate UCP1 expression and thus adaptive non-shivering or diet-induced thermogenesis in brown adipocytes, as well as the browning of the white adipose tissue (WAT) in C57BL/6 mice [67,68], Sprague-Dawley rats [69,70], and overweight humans [71].

Among the natural endogenous molecules with uncoupling properties, one can also cite the thyroid hormone T3. T3 regulates mitochondrial uncoupling by different mechanisms: (1) by sympathetic stimulation [61], (2) by increasing acylcarnitine production [61], thereby activating mitochondrial respiration/uncoupling, and (3) by directly stimulating the transcription of the *Ucp1* gene [37]. Of note, a rise in intracellular cAMP also induces the expression of thyroxine 5′-deiodinase in brown adipocytes and allows the generation of T3 close to these cells, which can form an amplification signal loop [37,72]. Interestingly, the uncoupler effect of T3 seems to require autophagy, at least partially. Indeed, hyperthyroidic Atg5KO mice are characterized by a lower body temperature and lower mitochondrial uncoupling than WT littermates [61]. Another example of a natural uncoupler is melatonin, the major active molecule secreted by the pineal gland. Although melatonin per se has no mitochondrial uncoupling effect, its metabolites 6-hydroxymelatonin and 5-methoxytryptamine are amphiphilic molecules capable of crossing biological membranes. A micromolar concentration is sufficient to significantly increase mitochondrial respiration in human MNT-1 melanoma cells [73].

### 2.3. Synthetic Mitochondrial Uncouplers

Carbonyl cyanide p-trifluoro-methoxyphenyl hydrazone (FCCP) and carbonylcyanide-3-chlorophenylhydrazone (CCCP) are the two classical mitochondrial uncouplers that are the most used in routine fundamental research. These molecules are lipophilic weak acids that act as protonophores. Due to their hydrophobic nature, these compounds can easily traffic across biological membranes and allow the protons to cross these membranes, mimicking the effect of UCP-1. However, the molecular mechanism is completely different (see Section 1: UCPs and ANTs). 

The non-specific effects of these protonophores are often underestimated. They can indeed affect any proton gradient (and even other ion gradient(s)) existing across any cell membrane. Already more than 20 years ago, Hollenbeck and colleagues showed that FCCP and CCCP stop mitochondrial movements in 3T3 cells and chicken neurites. This effect relies on a non-specific interaction with sulfhydryl groups of enzymes, such as dynein and myosin ATPases [74]. This class of compounds is not mitochondria-specific and can exert non-specific effects on other targets and organelles, including the plasma membrane. FCCP is well-known to induce plasma membrane depolarization in many cell types and species. For instance, FCCP induces H^+^ and Na^+^ currents across the plasma membrane in bovine aortic endothelial cells [75]. In isolated rat astrocytes, both DNP and FCCP induce Cl^-^ channel opening [76]. Exposure of mouse sensory neurons to FCCP leads to a rapid increase in intracellular/cytosolic Ca^2+^ concentration and to the secondary opening of Ca^2+^-activated K^+^ channels, causing a plasma membrane hyperpolarization [77]. At the opposite, DNP and FCCP (1 μM) lead to the depolarization of human glial cells (U-787CG), an effect independent of a rise in intracellular Ca^2+^ concentration [78]. In these two studies, the effect was not mediated by the drop in intracellular ATP concentration [77,78]. The effect of protonophoric mitochondrial uncouplers on plasma membrane is thus complex and at least partially dependent on species, cell type, and uncoupler used.

Beside these “classical” uncouplers, new molecules are constantly identified with more or less efficacy and untargeted/unspecific effects. For instance, BAM15 ((2-fluorophenyl)6-[(2-fluorophenyl)amino](1,2,5-oxadiazolo[3,4-e]pyrazin-5-yl)amine) is a novel mitochondria-specific protonophore uncoupler that possesses a similar potency to FCCP or DNP [45]. However, BAM15 has low or no effect on plasma membrane polarization, as seen in rat L6 myoblasts [45]. Interestingly, this compound also exhibits less cytotoxicity, suggesting that membrane depolarization should be taken into account in FCCP/CCCP-associated cell toxicity. More recently, another mitochondrial uncoupler was discovered, FR58P1 (a bromoalkyl ester of a hydroquinone derivative) [54]. As for BAM15, plasma membrane polarization is not affected by FR58P1 [54]. This compound seems to act only as a protonophore and does not inhibit mitochondrial complex I [54]. Moreover, and opposite of FCCP or CCCP (when used at similar concentrations), it exhibits only a slight decrease in the mitochondrial membrane potential. However, exposure of MDA-MB-231 cells to 30 μM FR58P1 still leads to a rapid decrease in intracellular ATP content, a drop in NAD^+^/NADH ratio, AMPK activation, and Sirt1 inhibition, suggesting that a mild mitochondrial uncoupling is sufficient to affect and reduce mitochondrial ATP production [54]. However, the ATP level is rapidly restored (4 h), probably because of a glycolytic shift, as indicated by a reduction in the expression of genes encoding products involved in OXPHOS (*cox-iv isoform 1*, *cyt c*, *atp5fa1*), mitochondrial ADP/ATP translocases (*ant2*, *ant3*), mitochondrial biogenesis (*pgc1a*, *nrf-1*), and glucose uptake (*glut1*, *glut4*) [54]. In agreement with its negative effects on the expression/activity of several key regulators of mitochondrial biogenesis, the abundance of mitochondria was also decreased in MDA-MB-231 cells exposed to this compound [54]. In summary, FR58P1 mildly uncouples mitochondria, leading to a mitochondrial clearance by mitophagy, activation of the cytoprotective AMPK/Sirt1 axis, and a glycolytic shift. Other mitochondria-specific uncouplers have also been reported: (1) MitoFluo, which is the result of the conjugation of a triphenylphosphonium cation to fluorescein, acting as a fluorescent uncoupler that accumulates preferentially in mitochondria [57,79]; (2) dodecyltriphenylphosphonium (C12TPP), which operates as a fatty acid anion carrier and facilitates fatty acid cycling across the membrane and thus mitochondria uncoupling [49]; (3) the Rhodamine 19 butyl ester C4R1, which seems to act as a mild mitochondrial uncoupler [60]; and (4) MitoPhotoDNP, which results from the fusion of DNP, the o-nitrobenzyl group (a photoactivable group), and triphenylphosphonium. Opposite of the other molecules, the latter compound can be activated by illumination at 355 nm, allowing the uncoupling of specific mitochondria in some specific cell areas [58].

Anesthetics is another class of compounds with some mitochondrial uncoupling properties. However, although many anesthetic agents can act as a mitochondrial uncoupler, their effects often differ in terms of mechanism of action. Although they were thought to act primarily as protonophores, the increase in mitochondrial ATPase activity induced by such compounds can be explained by direct effects on the activity or integrity of mitochondrial complexes [80]. As a consequence, mitochondrial respiration and mitochondrial proton leak are increased but without induction of a proper mitochondrial uncoupling. As an example, halothane, isoflurane, and sevoflurane inhibit mitochondrial complex I inhibition but not complexes II, III, and IV [55]. Divergent reports show that isoflurane could also act, at least partially, through inhibition of ATP synthase [81]. Bupivacaine, a local anesthetic agent, can act partially as a protonophore [48] but also presents an inhibitory effect on state 3-respiration by altering the mitochondrial proton pump stoichiometry [48]. Inactin, a thiobarbiturate often used for prolonged rat anesthesia, is also thought to possess some uncoupling properties. The effect of inactin is different according to the time of exposure. Short (5 min) or long (>1.5 h) incubation increases proton leak by UCP-2 or ANT activation, respectively [56]. However, inactin’s effect on mitochondrial respiration is also dependent on a direct effect on mitochondrial complexes. A concentration of 120 mg/kg is sufficient to inhibit complex I and induce ROS production, as observed on isolated kidney cortex mitochondria [56]. A similar effect was observed for isoflurane [81].

Finally, weak C-H acids, such as ortho-carborane (1,2-C_2_B_10_H_12_), were also shown to have global uncoupling properties, when used at concentrations comparable to FCCP (10 μM range) [82]. *N*-acyl amino acids also exhibit such properties, and a hydrolysis-resistant N-acyl amino acid analogue was recently identified. Its properties were validated both in vitro (C2C12 mouse myoblast cells) and in vivo (DIO mice) [59]. In endothelial cells (EA.hy926), CO-releasing molecules also present some uncoupling properties by activating mitochondrial large-conductance calcium-regulated potassium ion channels, suggesting that CO could regulate mitochondrial function [52].

### 2.4. Mitochondrial Uncouplers Affect Lysosomal Ion Homeostasis

Even though mitochondrial uncouplers can dissipate the mitochondrial proton gradient, they can also destabilize other ion gradients in other organelles. For instance, CCCP can interfere with lysosomal function and inhibit autophagic cargo degradation in both yeast and HeLa cells [83]. The positive effect of CCCP on lysosomal pH was demonstrated in single HeLa cells using the ratiometric pH-sensitive probe fluorescein-dextran [84]. The CCCP effect on lysosomal pH seems to be concentration- and time-dependent. Indeed, CCCP is known to rapidly activate mitochondrial degradation by mitophagy [83,85] via a mechanism discussed later in this review, and it has been suggested that this could be due to the generation of a negative lysosomal membrane potential (by decreasing the lysosomal pH gradient without affecting ion counter-gradients) [83]. Of note, other authors have reported conflicting results and found no significant effect on lysosomal function and an almost complete loss of mitochondrial mass in HeLa cells exposed to the uncoupler at 10 μM for 48 h [85]. These divergences might be explained by the techniques used to quantify the mitochondrial population [83]. Though many authors use fluorescent probes that accumulate in mitochondria, the use of such techniques can lead to the observation of an almost complete clearance of mitochondria. However, some structures positive for TOMM20 (Translocase of Outer Mitochondrial Membrane 20) were observed in HeLa cells exposed to CCCP (20 μM, 24 h), suggesting that mitochondrial morphology and structure are severely affected by the mitochondrial uncoupler [83] (which limits their detection using mitochondria-specific probes), and that mitochondrial clearance induced by CCCP is not complete. Of note, some specific “uncoupling” of the lysosome could also be achieved by inhibiting the lysosomal H+ V-ATPase. For instance, the exposure of HeLa cells to 2 µM concanamycin A leads to a rapid increase in lysosomal pH (raising from 5.5 to 7.0 within 10 min) [84].

## 3. Cellular Responses to Mitochondrial Uncoupling

**Figure 1:**
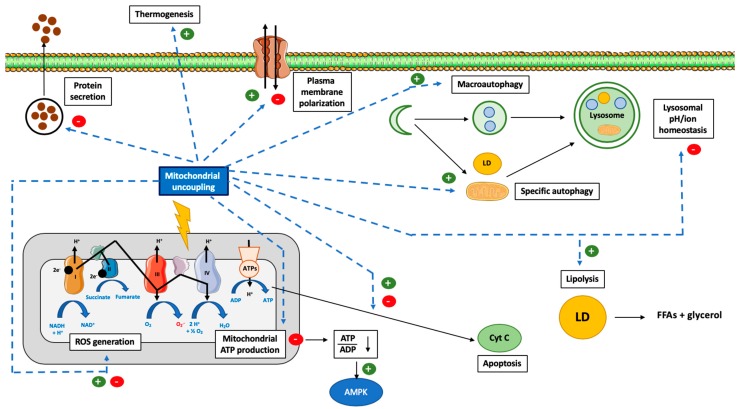
Overview of the cellular consequences of mitochondrial uncoupling. Abbreviations: ADP (Adenosine DiPhosphate), AMPK (Adenosine Monophosphate-Activated Protein Kinase), ATP (Adenosine Triphosphate), ATPs (ATPsynthase), CytC (Cytochrome C), FFA (Free Fatty Acid), LD (Lipid Droplet), ROS (Reactive Oxygen Species), NAD (Nicotinamide Adenine Dinucleotide). Induction of mitochondrial uncoupling by use of synthetic or natural uncoupling agents or by activating dedicated proteins, such as UCPs or ANTs, can trigger several cellular mechanisms. The first effect, and the most well-known, is the inhibition of mitochondrial ATP production by ATP synthase due to the dissipation of the mitochondrial proton gradient. This energy will be dissipated as heat (thermogenesis). The decrease in cytosolic ATP will be sensed by AMPK and lead to its activation. In addition, the decrease in mitochondrial potential membrane will also modify ROS generation. In order to cope with this energy loss, autophagy will also be triggered (both bulk and specific forms of autophagy). Lipid droplets (LD) will be degraded by a form of autophagy and help to fuel the cell with lipids. Loss of the mitochondrial potential membrane will also allow the identification of these dysfunctional mitochondria by autophagy (mitophagy). Mitochondrial uncoupling can also help to protect cells against cell death and apoptosis but can also promote it, according to the cell type, mitochondrial uncoupler and mitochondrial uncoupling intensity considered. Finally, the use of non-specific mitochondrial uncouplers, such as FCCP or DNP, could alter the homeostasis of several ions, such as Ca^2+^, Na^+^ and K^+^ (at the cytosolic, mitochondria, or lysosomal levels), which will lead to a decrease in protein secretion and plasma membrane (de)polarization, respectively.

It can be easily understood that the activation or induction of mitochondrial uncoupling will lead to the activation of cellular mechanisms/responses in order to cope with induced stress and/or to regulate this process. For instance, a severe mitochondrial uncoupling can lead to rapid cellular ATP depletion and, if the stress persists, to the triggering of other cellular mechanisms, such as cell death. In this part of the review, we will cover how cells can respond to mitochondrial uncoupling. The cellular responses to mitochondrial uncoupling are also depicted in Figure 1.

### 3.1. Autophagy

Three forms of autophagy have been described: Macroautophagy, microautophagy, and chaperone-mediated autophagy (CMA) [86,87]. CMA seems to only exist in mammals, while both macro- and micro-autophagy have been reported in mammals and yeast [86,87]. Macroautophagy can be defined as the capture of cytosolic material (such as organelles, accumulated debris, invasive microbes, proteins, or organelles) by autophagosomes [86]. Microautophagy consists in the direct capture of material by lysosomal invaginations [88]. Both macroautophagy and microautophagy processes can be either non-specific or targeted (against mitochondria, ribosomes, or lipid droplets, or other subcellular structures). We will not discuss here the molecular actors of autophagy, but we invite the reader to continue reading with a dedicated review [89].

Mitochondrial uncoupling has often been associated with autophagy activation, and more specifically, with mitophagy (specific degradation of mitochondria by macroautophagy) (Figure 1). Indeed, the mild uncoupling of oxidative phosphorylation by various mitochondria-targeted penetrating cations may contribute to their reported therapeutic effects via the induction of both autophagy and mitochondria-selective mitophagy [90]. In this section, we will review data available regarding the interconnection between these two processes, with details regarding the specificity of the regulated autophagy form, when possible.

The positive effect of mitochondrial uncoupling on autophagy has been established in many cellular and animal models, including adipocytes [91], MEFs [92], HeLa cells [93], HepG2 cells [90], SH-SY5Y neuroblastoma cells [93], or C57BL6 mice [94]. However, mitochondrial uncouplers can elicit different cellular responses according to the cell types. For instance, low doses of CCCP (5 μM) or C12TPP (0.2 nM) do not upregulate macroautophagy in human EAhy926 endothelial cells but still induce an anti-inflammatory response, including reduction in the expression of E-selectin, ICAM1, and VCAM1 and in the adhesion of neutrophils to endothelium induced by TNFα (Tumor Necrosis Factor α) [95]. However, only the antioxidant action of the mitochondrial uncouplers seems to be responsible for this effect [95].

Besides its role in macroautophagy, mild mitochondrial uncoupling is also linked to lipid metabolism and more specifically to lipid disposal by lipophagy. We recently showed that FCCP triggers a form of autophagy in 3T3-L1 murine white adipocytes (increase in LC3-II protein abundance, reduction in p62) [91]. In this model, a low concentration (0.5 μM) in this mitochondrial uncoupler is sufficient to reduce adipocyte triacylglycerol (TAG) content, resulting from a lipolysis activation that is independent of the classical cytosolic lipases HSL (Hormone Sensitive Lipase) and ATGL (Adipose Triglyceride Lipase) [91]. This phenomenon seems to be dependent, at least partially, on a form of autophagy independent of Atg5/7 [91]. Lysosomal poisoning also prevents FCCP-induced TAG content decrease, suggesting a role for autophagy in this process [91]. Our results are in accordance with the increase in glycerol release found in 3T3-L1 murine adipocytes overexpressing UCP-1 [96].

Besides their role in white adipocytes, autophagy and lysosomes play a major role in thermogenesis regulation in brown adipocytes, a process under the control of UCP-1. Indeed, mice deficient for LAL (Lysosomal Acid Lipase, lysosomal lipase) exhibit BAT with lysosome and lipid droplet accumulation, reduced UCP-1 expression, and mitochondrial dysfunction [97]. This finding clearly indicates the crucial role of lysosome in TAG degradation to aliment thermogenesis and mitochondrial uncoupling. Moreover, UCP-1 expression and lysosomal function seem to also be tightly interconnected. Indeed, the differentiation of 3T3-L1 cells into white adipocytes is accompanied by an increase in autophagy and a parallel decrease in UCP-1 expression. The transcription factors FoxO1 (Forkhead box protein O1) and TFEB (Transcription Factor EB) interact with each other and regulate directly and negatively UCP-1 expression [98]. 

An interconnection between autophagy and mitochondrial uncoupling was also found in cardiomyocytes and neurons. Indeed, the mitochondrial uncoupler cloxyquin (5-chloro-8-hydroxyquinoline) protects mouse cardiomyocytes against ischemia-reperfusion, an effect requiring autophagy [99]. Mechanistically, mitochondrial uncoupling induced by cloxyquin is accompanied by an increase in macroautophagy characterized by an increase in LC3-II abundance and in autophagosome number [99]. Mitochondrial uncoupling-induced macroautophagy seems to be necessary for ischemia-reperfusion protection, as an autophagy inhibitor (chloroquine) completely abolishes the cloxyquin effect [99]. The mechanistic link between autophagy and mitochondrial uncoupling was, however, not elucidated [99]. In primary murine mesencephalic cells, the autophagy inducer rapamycin protects the cells against rotenone-induced cell death [100]. Interestingly, rapamycin treatment is accompanied by an increase in mitochondrial potential membrane [100], suggesting, again, a link between mitochondrial (un)coupling and autophagy. To date, the mechanism linking mitochondrial uncoupling and autophagy is not clear but is likely to include regulation of mTOR signaling. Indeed, mice treated with DNP present a decrease in mTOR activity, in the insulin-PI3K-MAPK signaling pathways, and a stimulation of the Ca^2+^-CREB axis accompanied by an increase in autophagy [94]. 

### 3.2. Mitophagy

The most well-known effect of mitochondrial depolarization induced by protonophores such as FCCP or CCCP is the promotion of mitochondrial degradation by autophagy (mitophagy). This phenomenon was observed in several cellular models, including HeLa cells, SH-SY5Y neuroblastoma cells, mouse cells [93], reticulocytes, or porcine oocytes [101]. In the context of mitochondrial uncoupling, this mechanism is mainly dependent on the recruitment of the serine/threonine kinase PINK-1 (Pentaerythritol Tetranitrate–Induced Kinase 1) and the E3 ubiquitin ligase Parkin. As a consequence, ubiquitinated proteins are accumulated at the outer mitochondrial membrane (to form a protein complex >500 kDa [102]), which will be recognized by adaptor proteins (such as VDAC1 (Voltage-Dependent Anion Chanel 1), HDAC6 (Histone DeAcetylase 6) and p62/SQSTM1 (Sequestosome 1)) [93,103,104]. Finally, the autophagy machinery, such as LC3, will also be recruited, autophagosomes will be formed, and dysfunctional mitochondria will be degraded. The molecular mechanisms of mitophagy were reviewed in detail elsewhere and will not be discussed here [105,106].

Mitophagy is a complex process triggered by many pathophysiological conditions, such as reticulocyte maturation [107], hypoxia [108], cold exposure in brown adipocytes [109], mitochondrial DNA damage [110] or sperm mitochondria removal after fertilization [111], and mitochondrial uncoupling. A mild mitochondrial uncoupling as induced by 3-nitropropionic acid (–30% in TMRM staining, a fluorescent probe used to assess mitochondrial potential membrane) is sufficient to trigger massive mitophagy in mouse neurons. This suggests that mitophagy is either a very sensitive process or that mitochondrial depolarization is not always a complete requisite for mitophagy [112]. Another example is the parkinsonian neurotoxin 6-OHDA (6-hydroxydopamine), which also induces a mild mitochondrial uncoupling (10%) without triggering mitophagy in neurons and SH-SY5Y cells [113]. Finally, FCCP-induced autophagy also seems to be independent from mitochondrial depolarization and could instead rely on a cytosol acidification [114]. Nigericin stimulates mitophagy through the same mechanism [114]. In this review, we will only discuss the mechanisms clearly identified as triggered by mitochondrial uncoupling.

The recognition of depolarized mitochondria by mitophagy machinery is performed by different proteins. First, Parkin is recruited to the outer mitochondrial membrane. The Parkin function is thought to prevent mitochondrial 3D conformational changes by causing mitofusin ubiquitination and degradation [115]. Its binding seems to be regulated by PTENα (Phosphatase and Tensin protein α), a tumor suppressor gene encoding a phosphatase that plays a major role in the cell cycle but also in mitochondrial metabolism (regulation of cytochrome c oxidase activity and mitochondrial ATP production) [116]. As an illustration of its importance, PTENα KO mice exhibit the accumulation of dysfunctional mitochondria in cardiomyocytes [117]. Importantly, loss of PTENα is sufficient to impair degradation of CCCP-depolarized mitochondria in mouse cardiomyocytes [117], suggesting an impairment of the recognition of damaged mitochondria by mitophagy machinery. In addition to PTENα, UBXD1 (UBX Domain Containing Protein 1), a member of the p97 adapter protein family, also helps to recognize depolarized mitochondria by its C-terminal UBX domain and in a Parkin-dependent manner. Once bound, it recruits its cofactor, p97. Altogether, they stimulate autophagosome formation and mitochondria degradation [118]. Of note, some forms of mitophagy triggered by mitochondrial uncoupling do not seem to require Parkin binding to mitochondria [106,119,120]. In addition, mitochondrial uncoupling itself seems to decrease Parkin protein abundance by increasing miR-181a abundance in SH-SY5Y cells (human neuroblastoma cells) and A172 cells (human glioblastoma cells) [121]. Interestingly, the opposite was also observed: The overexpression of miR-181a is sufficient to alter the mitochondrial potential membrane and to induce mitophagy [116]. As for Parkin, PINK1-independent forms of mitophagy have been reported. Indeed, in PINK1^-/-^ murine cardiomyocytes, mitochondrial depolarization induced by FCCP is still able to induce Parkin1 recruitment and mitophagy [122].

Mitochondrial Sirtuins form a group of 3 proteins, namely SIRT3, SIRT4, and SIRT5, involved in metabolic homeostasis, protein acetylation, antioxidant defenses [123,124] and mitophagy regulation. Although the precise enzymatic activity of SIRT4 is still unclear [125], its moderate overexpression leads to an increase in the inner-membrane-bound long form of the OPA-1 GTPase (L-OPA1) and to an increase in mitochondrial fusion [126]. The overexpression of SIRT4 is also sufficient to counteract the effect of CCCP in HEK293 cells [126], suggesting that both the increase in mitochondrial fission and the decrease in mitochondrial fission are a prerequisite for mitochondrial uncoupling-induced mitophagy. This hypothesis is further supported by the fact that the presence of L-OPA1 at the inner mitochondrial membrane is sufficient to delay mitophagy [127]. By binding both OPA1 and Drp1, FUNDC1 (FUN14 Domain Containing 1) could regulate the equilibrium between mitochondrial fusion and fission [128]. FCCP mitochondrial depolarization could induce the disassembly of the FUNDC1-OPA1 complex, Drp1 binding to mitochondria, and mitophagy induction [128]. 

Another crucial factor for mitochondrial uncoupling-induced mitophagy is reactive oxygen species (ROS) generation. A short exposure of HeLa cells to CCCP (2 h) is sufficient to trigger the mitochondrial translocation of Parkin—but when CCCP is removed, mitophagy does not proceed further, suggesting that another actor is required [119]. However, if H_2_O_2_ is provided immediately upon CCCP removal, mitophagy continues, suggesting the involvement of ROS to complete the biological process [119]. In addition, the superoxide anion would be an important actor of this process. Indeed, diethyldithiocarbamate, a SOD (Superoxide Dismutase) inhibitor, promotes mitophagy in HeLa cells [119]. In accordance with the ROS theory, another study reported that, in 293T HEK (Human Embryonic Kidney) and HeLa cells, peroxiredoxin 6 (PRDX6) is recruited shortly after exposure to FCCP, an event eventually leading to the recruitment of Parkin [129].

In terms of cellular signaling, mitochondrial uncoupling-induced mitophagy seems to rely mainly on mTORC1 inhibition, as TSC2^-/-^ MEFs still exhibit mitophagy activation in the presence of mitochondrial uncouplers [130]. CCCP-induced mitophagy in mammalian cells seems to also be based, at least partially, on an unconventional form of autophagy independent of Atg5/7/12 but requires Erk2/p38 signaling [131]. The role of AMPK activation, in response to a decrease in the ATP/AMP ratio caused by mitochondrial uncoupling as a requirement for mitophagy activation, has been questioned as CCCP is able to inhibit the activity of mTORC1 independently of AMPK [92]. In AMPKα1/α2 double KO MEFs, this protonophore still elicits autophagosome formation and mitochondria disposal [92]. Moreover, there was no difference in term of colocalization between mitochondria and autophagosomes in WT and double KO MEFs [92]. 

Once the stress is over, a molecular mechanism must exist to repair or eliminate the accumulated damages. If mitophagy helps to dispose of dysfunctional mitochondria, then mechanisms should exist in order to increase, in parallel, the lysosome number (to ensure complete mitochondria degradation) and mitochondrial population (to restore it after its degradation). As expected, shortly after mitophagy initiation by CCCP, the transcription factors Nrf2 (Nuclear factor erythroid 2-related factor 2) and TFEB are both translocated to the nucleus [132]. Altogether, they stimulate mitochondrial [133] and lysosomal biogenesis, respectively [132]. In addition, TFEB controls the expression of the Atg family [134,135]. Regarding the activation of Nrf2 after CCCP treatment, it seems to rely on a p62-dependent degradation of the Nrf2-inhibitor protein Keap1 [136]. Such coordination between mitochondrial disposal and biogenesis was also observed in cold-induced mitophagy in brown adipocytes [109].

### 3.3. Reactive Oxygen Species Production

**Figure 2:**
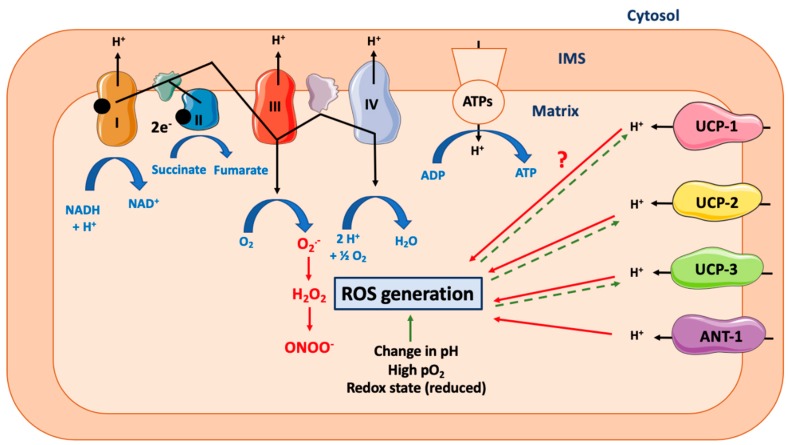
Effect of mitochondrial uncoupling proteins on mitochondrial ROS generation. Abbreviations: ANT-1 (Adenine Nucleotide Transferase-1), ATPs (ATPsynthase), IMS (Inter Membrane Space of Mitochondria), ROS (Reactive Oxygen Species), UCP-1 (Uncoupling Protein-1). Mitochondrial ATP production relies on the coupling between the proton gradient on either side of the inner mitochondrial membrane and the use of this proton-motive force to feed the mitochondrial ATP synthase complex. However, this coupling is not complete and could lead to the production of ROS. Many different ROS can be produced by the mitochondria, including superoxide anion (O_2_^•−^), which is dismuted into hydrogen peroxide (H_2_O_2_), and could generate peroxinitrite (ONOO^-^). Many other parameters can increase the ROS production rate, including change in cytosolic or mitochondrial pH, the redox cellular state, or high local O_2_ availability. UCP-1 is a protein specialized in proton transport across the inner mitochondrial membrane. By uncoupling the mitochondrial respiration, it decreases the ROS generation rate. Of note, this effect is likely to be dependent on cell type and species. At the opposite, the role of UCP-2 and UCP-3, two other members of UCP protein family but with a less defined proton transport capacity, is clearer and consists in ROS reduction. Activation of ANT-1 also leads to a decrease in ROS production. Of note, high ROS production will also lead to a cellular adaptation and to an increase in UCPs expression (dashed line).

According to Mitchell’s chemiosmotic theory, mitochondrial ATP production relies on the coupling between the proton gradient on either side of the inner mitochondrial membrane and the use of this proton-motive force to feed the mitochondrial ATP synthase complex [1]. However, this coupling is not complete and leads in fine to the production of ROS, a process called electron leak [3] (Figure 2). Many different ROS can be produced by the mitochondria, including the primary ROS superoxide anion (O_2_^•−^), which is dismuted into hydrogen peroxide (H_2_O_2_), and could generate peroxinitrite (ONOO^−^). The reactive oxygen release can account for 0.1–0.2% of O_2_ consumed, as measured in different rat tissues [137]. Of note, although ROS production is often seen as a result of mitochondrial potential membrane disruption, many other parameters also impact the ROS production rate, including (1) the mitochondrial/cytosol pH difference or any pH disturbance leading to ROS production [138], (2) the redox cellular state (reduced state favors mitochondrial ROS production) [139,140], or (3) local O_2_ availability (hyperoxia increases mitochondrial ROS production) [140,141,142].

Based on the simple model described above, the induction of mitochondrial uncoupling would theoretically lead to a decrease in ROS production (Figure 2). This hypothesis was confirmed in many models, including, for example, isolated rat and pigeon liver mitochondria (decrease in H_2_O_2_) [143], human monoblastic ML-1 cells (decrease in O_2_^•(−)^ and ONOO^−^), and rat brains, hearts, kidneys, livers, and skeletal muscles [137]. However, despite these pieces of evidence, the putative effects of mitochondrial uncouplers on ROS generation are still debated [14,144]. Moreover, as mentioned before, many cellular parameters could influence this process, such as cytosolic pH or pO_2_, as well as the nature and origin of the mitochondrial uncoupling effect. Altogether, these elements could eventually lead to conflicting results. For instance, while both FCCP and CCCP increase O_2_^•−^ production in rat vascular smooth muscle cells [145], BAM15, a mitochondria-specific uncoupler, does not change ROS production in this cell type despite efficient induction of mitochondrial uncoupling [45]. The state activity of the different mitochondrial complexes also influences ROS production regulation in response to mitochondrial uncouplers. Indeed, FCCP reduces O_2_^•−^ production in ML-1 cells when used in combination with antimycin A (an inhibitor of complex III) [146], while inhibition of both complexes I and III further stimulates CCCP-induced mitochondrial H_2_O_2_ production [137].

The activation of UCP-1 and its potential effect on mitochondrial ROS production are also unclear [144]. Mitochondrial H_2_O_2_ production is decreased in cold-stimulated brown adipose tissue mitochondria of wild-type C57BL/6J mice when compared to UCP1-ablated littermates [147,148]. However, the ablation of *Ucp1* expression was shown to increase H_2_O_2_ production in murine brown adipocytes [149]. UCP-1 could thus protect brown adipocytes against cold-induced ROS production. However, UCP1 deletion is probably not the best model to study UCP1 function. Indeed, such a condition could lead to (1) mitochondrial dysfunction (decreased mitochondrial calcium buffering capacity and increased sensitivity to mitochondrial permeability transition opening induced by ROS) and (2) activation of the innate immune signaling and cell death in brown adipocytes [150]. In addition, brown adipocytes have also been shown to produce H_2_O_2_ in a UCP-1-independent manner, a phenomenon dependent on acyl-CoA dehydrogenase and electron transferring flavoprotein-ubiquinone reductase [151]. Of note, high levels of H_2_O_2_ and O_2_^•−^ were reported in cold-stimulated murine brown adipocytes in vivo, suggesting that UCP-1 does not totally prevent these cells from producing ROS [152]. Interestingly, ROS, in turn, can also modify the activity of UCP-1 and affect mitochondrial uncoupling. Indeed, ROS induce sulfonylation of UCP-1 on Cys253, which sensitizes UCP-1 activity to adrenergic activation and promotes in vivo thermogenesis [152].

The role of other UCP members (especially UCP-2 and -3) on ROS production is more documented and was recently reviewed [153]. These UCP members are known to alleviate oxidative stress by decreasing mitochondrial ROS production [153]. The overexpression of these UCPs also protects against excessive superoxide production and oxidative damage observed in pathological situations, such as stroke and ischemia/reperfusion [153]. As mentioned before, these UCPs do not possess a well-defined uncoupling property and therefore their role on ROS production is likely to be independent of mitochondrial uncoupling. In accordance with this hypothesis, UCP-2 and -3 protect L6 muscle cells against mitochondrial ROS production without inducing mitochondrial uncoupling [154]. In primary rat neuron cultures, exposition to β-amyloid leads to the overexpression of UCP-2 that reduces ROS generated in this condition and limits toxicity [155].

In addition to UCP-1, ANT-1 activity can also regulate ROS production. Indeed, skeletal muscles from ANT-1-deficient mice exhibit a 3- to 4-fold increase in H_2_O_2_ levels [156]. Physiological repression of ANT-1 expression also leads to an increase in H_2_O_2_ production in T98G human glioblastoma cells and rat cortical neurons [157]. Indeed, the activation of NFκB signaling in cells exposed to TNFα leads to reduced ANT-1 mRNA and protein expression and to an increase in H_2_O_2_ generation [157]. Beta-lapachone, a quinone isolated from the lapacho tree (*Tabebuia avellanedae*), can also be specifically accumulated in mitochondria, where it induces ROS generation [158]. In fine, excessive ROS production damages mitochondria and induces secondary mitochondrial respiration uncoupling [158].

### 3.4. Protein Secretion

As mentioned before, the use of mitochondrial uncouplers, such as FCCP or CCCP, can induce non-specific effects independent of mitochondria uncoupling. Effects on Golgi and post-Golgi compartments were suggested more than 30 years ago [159]. For instance, FCCP inhibits the transport of immunoglobulins from the Golgi apparatus to the plasma membrane by 80% [159]. To explain this effect, it was suggested that mitochondrial uncoupling, by decreasing ATP content, could slow down the energy-demanding steps in protein secretion [160]. Mitochondrial uncouplers are thus likely to interfere with protein secretion (Figure 1).

The effects of uncouplers on protein secretion seem to be complex, as illustrated here for the beta-amyloid precursor protein (APP), a protein playing a key role in the etiology of Alzheimer’s disease. APP protein is cleaved by α-secretase within the Aβ sequence and then released in the extracellular space as APPsα. An FCCP concentration that does not impact ATP content is still sufficient to decrease APP secretion in human embryonic kidney 293T cells [161]. This observation suggests that either a low mitochondrial uncoupling or proton gradient dissipation (for instance, in lysosome or in the autophagosome pathway) might play a role in APP processing.

Mitochondrial uncoupling could also affect Ca^2+^ homeostasis. While calcium is essential for the activity of three enzymes regulating the TCA/Krebs cycle (pyruvate dehydrogenase, α-ketoglutarate dehydrogenase, and isocitrate dehydrogenase), mitochondria are also a key actor involved in Ca^2+^ homeostasis, as they act as a cellular reservoir with a calcium buffering capacity [162]. Calcium is essential for the regulation of the release of several hormones in cell types such as pancreatic beta and alpha cells or pheochromocytes (chromaffin cells). As a mitochondrial potential membrane is required for Ca^2+^ entry into mitochondria [163], mitochondrial uncoupling will interfere with Ca^2+^ homeostasis and with Ca^2+^-mediated protein exocytosis. In accordance with this theory, use of CCCP or FCCP decreases the Ca^2+^ buffering capacity of mitochondria and increases global exocytosis in bovine [164,165] and rat chromaffin cells [166] but not in the same cell type in mice [167], suggesting species differences. In the rat insulinoma cell line INS-1E and in primary mouse and rat pancreatic beta cells, use of FCCP/CCCP inhibits glucose-stimulated insulin release (GSIS) [168,169,170]. However, insulin release in INS-1 832/13 cells, another rat insulinoma cell line, seems to be resistant to FCCP-induced proton leak [171]. 

Although mitochondrial uncouplers can affect protein secretion, the precise effect of a pure mitochondrial uncoupling, as catalyzed by UCP-1 for instance, on protein secretion has been poorly studied. In 3T3-L1 adipocytes, the ectopic overexpression of UCP-1 does not significantly affect leptin secretion [96], while in mice, *Ucp1* deletion leads to an increase in the expression of leptin and MCP-1 (Monocyte Chemoattractant Protein), a chemokine regulating the migration and infiltration of macrophages [172]. However, this effect is likely to be due to the WAT lipodystrophy observed in these mice, which increases local inflammation and affects leptin secretion [172]. In other cell types, UCP-1 overexpression seems to have a direct impact on protein secretion. For instance, in rat insulinoma cells, the overexpression of UCP-1 prevents glucose-induced ATP increase, thereby limiting their glucose-stimulated insulin release capacity [173]. In addition, the specific overexpression of UCP-1 in mice skeletal muscle cells not only induces mitochondrial uncoupling but also triggers the ISR (Integrated Stress Response) pathway, which is characterized by the phosphorylation of eIF2α and the activation of ATF4 [174] accompanied by an increase in Fgf21 secretion, which in turn induces WAT browning [174].

### 3.5. Cell Death

Mitochondrial uncouplers can be cytotoxic, especially at high concentrations, an effect dependent, at least partly, on the drop in the ATP level and on plasma membrane/lysosomal depolarization/permeabilization. However, depending on the concentration used, mitochondrial uncoupling could also help to protect cells against cell death (mitohormetic response [175], Figure 1). For instance, pretreatment with low doses of FCCP (100 nM) protects cardiomyocytes against ischemia stress [176]. At this concentration, an increase in mitochondrial respiration is observed, but there is no change in mitochondrial membrane potential [176]. The protective effect of low doses of FCCP seems to be dependent on ROS and is completely inhibited by *N*-acetyl cysteine, an antioxidant molecule active in the cytosol [177]. Low concentrations (1–5 μM) of FCCP also promote cancer cell survival in response to topoisomerase inhibitors [178] or other chemotherapeutic agents, such as gemcitabine [179], reducing apoptosis triggered by these molecules. In accordance with the ROS theory, the FCCP effect was mimicked by UCP-2 overexpression [178,179,180]. Low concentrations of DNP can also protect neurons against anoxia by maintaining the mitochondrial membrane potential and by preventing mitochondrial permeability transition pore opening, as seen in newborn rats [181].

The role of UCP-1 in the control of cell death and apoptosis has been poorly studied. Moreover, apoptosis is often an indirect effect of UCP-1 activation. For instance, injection of LPS in mice leads to an increase in hepatic UCP-1 expression and increased cell apoptosis [182]. Similar results were obtained in the liver tissue of sepsis patients [182]. However, the direct link between UCP-1 activity and apoptosis induction was difficult to establish. In thymus, UCP-1 would play a role in thymocyte selection. Indeed, *Ucp1* KO mice exhibit less spleen cells and an increase in CD4/CD8 double positive cell numbers in thymus, suggesting decreased apoptosis in cells from UCP-1 KO mice [183]. 

Contrary to UCP-1, the effect of the overexpression of ANT-1 (but not ANT2 [184]) is well-known to promote apoptosis. This effect was observed in HeLa, human embryonic kidney cell 293T, baby hamster kidney cells, and rat neonatal ventricular myocytes [184,185,186]. Interestingly, point mutations inhibiting the ANT-1 uncoupling property do not protect against ANT-1-mediated apoptosis in 293T cells, suggesting that this effect is independent of mitochondrial uncoupling [185]. However, these results are challenged by experiments performed on mitochondria isolated from human leukemia HL-60 cells exposed to ANT-1 inhibitors, such as MT-21 or atractyloside, showing a stimulation of apoptosis and cytochrome c release [187]. Mechanistically, ANT-1-mediated cell apoptosis is dependent on cytochrome c release and mitochondrial transition pore opening [184,185,186]. This phenomenon also seems to be dependent on the recruitment of the IκBα-NFκB complex to mitochondria, decreasing the nuclear abundance of NFκB and the transcription of its target anti-apoptotic genes [184]. It was also suggested that, in some models, such as in rat myocytes, the activation of apoptosis could be independent from cytochrome c release and mitochondrial transition pore opening but may be due to an important ROS generation [186]. 

### 3.6. Physical Exercise

**Figure 3:**
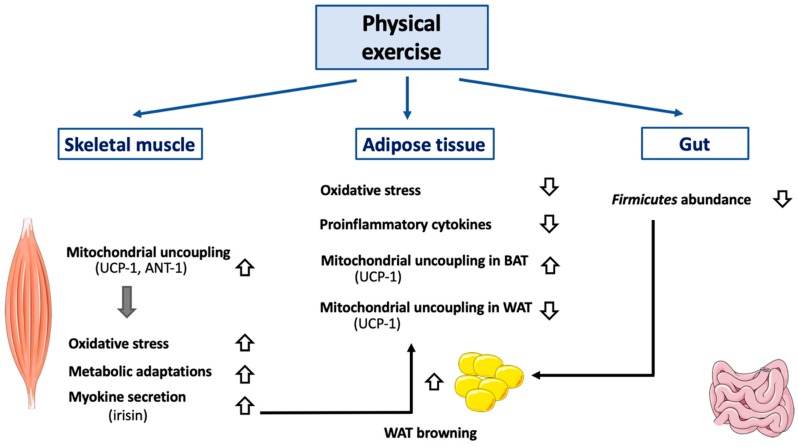
Effect of exercise on mitochondrial uncoupling and peripheral organs. Abbreviations: ANT-1 (Adenine Nucletotide Transferase-1), BAT (Brown Adipose Tissue), UCP-1 (Uncoupling Protein-1), WAT (White Adipose Tissue). Different kinds of physical exercise (acute and chronic) trigger mitochondrial uncoupling in skeletal muscle, by activating and by increasing both UCP-1 and ANT-1 expression. Of note, molecular mechanisms triggered differ according to the species considered and are not yet entirely defined. Activation of mitochondrial uncoupling in skeletal muscle also reduces oxidative stress and induces mitochondrial adaptations (including increase in mitochondrial mass, increase in respiratory chain component abundance). Physical exercise also has an impact on adipose tissues. In BAT, mitochondrial uncoupling will be activated (UCP-1 dependent), while it will be attenuated in WAT. Browning of WAT will also be induced, an effect dependent, at least partially, on irisin secretion by skeletal muscle. An association between decreased *Firmicutes* level in gastrointestinal tract (associated with physical exercise) and WAT browning has also been observed. In case of sustained physical exercise, adipose tissue mass will decrease which will alleviate both oxidative stress and secretion of proinflammatory cytokines.

Several pieces of evidence show that a tight interconnection *does* exist between physical exercise and mitochondrial uncoupling. For an excellent review describing the many beneficial effects of physical exercise on mitochondria, cell signaling, and life span/aging, see [188]. First, physical exercise by itself directly reduces proton leak and ROS production and increases mitochondrial membrane potential, suggesting an improvement of mitochondrial coupling (Figure 3). This effect was first noticed in Long-Evans Tokushima Otsuka rats, an animal model of non-insulin-dependent diabetes mellitus [189]. Other research groups reported opposite results (increase in H_2_O_2_ production, decrease in mitochondrial coupling) in endurance-trained Wistar rats [190], suggesting possible differences between animal strains and/or differential effects of different kinds of exercise trainings (acute, chronic, severity level, etc.) A rise in mitochondrial oxidative stress during exercise seems to be required in order to lead to metabolic mitochondrial adaptations. Indeed, *SOD2* KO mice cannot compensate for increased oxidative stress during exercise and fail to increase maximal work capacity, mitochondrial enzyme activity, and mtDNA copy number [191]. In humans, physical exercise increases total mitochondrial mass and uncoupling in skeletal muscular cells [192]. Physical inactivity has the opposite effect and reduces mitochondrial uncoupling in skeletal muscles of humans [193]. However, this effect does not seem to be dependent on a decrease in UCP-1 expression but might result in ANT-1 downregulation [193]. Physical exercise would thus be beneficial, at least partly, by augmenting the mitochondrial uncoupling-driven thermogenesis (and thus energy expenditure). As an example, acute physical exercise leads to an increase in BAT UCP-1 protein expression in HFD-treated (High Fat Diet) Swiss mice [194] and ICR (Institute of Cancer Research) mice [195]. The importance of UCP-1 expression was illustrated in a recent study performed in *Ucp1*^−/−^ C57BL/6J female mice, showing an increased sensitivity to Western diet [172]. The acute exercise effect on thermogenesis can be explained by a potentiation of leptin-induced hypothalamic ERK1/2 phosphorylation, which stimulates BAT thermogenic function [194]. In WAT, physical exercise seems to promote the opposite effect. Indeed, physical exercise would reduce UCP-1 and PGC-1α (Peroxisome Proliferator-Activated Receptor γ Coactivator 1α) protein expression in sub-cutaneous WAT of HFD-treated C57BL/6 mice [196]. Global content in mitochondrial protein was also reduced in response to physical exercise in these mice [196]. Of note, leptin signaling by itself could help to protect the cells from mitochondrial uncoupling. Once bound, leptin induces the proteolysis of its receptor, leading to the generation of a fragment named Leptin Receptor IntraCellular Domain (LR-ICD). Interestingly, LR-ICD can interact with SOCS6 (Suppressor of Cytokine Signaling 6) and be recruited to the mitochondrial surface [197]. LR-ICD almost totally prevents the CCCP-induced mitochondrial depolarization and subsequent mitophagy. Though it remains to be demonstrated that this phenomenon does exist in cell types other than HeLa cells, LR-ICD release could also prevent severe mitochondrial uncoupling in BAT. Of importance, physical exercise has an obvious effect on skeletal muscles but also impacts other cell types, such as neurons. Indeed, in mice, acute exercise increases hypothalamic sphingine-1-phosphate (S1P), S1P receptor 1 expression, and STAT3 phosphorylation—events that stimulate, in fine, UCP-1-dependent BAT thermogenesis [198].

Chronic exercise would have a similar effect on UCP-1 expression, as seen in Wistar rats [194,199], and would be accompanied by an increase in carnitine palmitoyltransferase II, the mitochondrial F1 ATP synthase α-chain, and mitochondrial malate dehydrogenase 2. In terms of cell signaling, chronic exercise increases SIRT1 expression, PGC1α, and activation of AMPK by phosphorylation (Thr172/183) in the skeletal muscles of rats [200]. Intriguingly, physical exercise also protects mice against cold-induced weight loss, but UCP-1, and thus mitochondrial uncoupling, does not seem to be involved in the process [201]. Underlining the importance of AMPK’s role in exercise-induced metabolic adaptations, β1β2M-KO mice are characterized by decreased physical activity and a reduction in skeletal muscle mitochondrial content after treadmill training [202].

Physical exercise also stimulates the secretion of peptides or hormones by skeletal muscle cells, namely the myokines. One of the most important myokines is irisin. This polypeptide of 112 amino acid residues results from the cleavage of the extracellular domain of a protein named fibronectin type III domain-containing protein 5 (FNDC5) [203]. Irisin is mainly secreted by skeletal muscle cells, although some secretion was also reported in WAT [204] and, to a lesser extent, in BAT [204,205]. The main and well-described function of irisin is to induce WAT browning and thus UCP-1-dependent mitochondrial uncoupling [206]. This hormone also increases glucose uptake in murine myocytes but decreases the expression of genes encoding enzymes involved in liver gluconeogenesis, such as PEPCK (PhosphoEnolPyruvate CarboxyKinase) and G6Pase (Glucose-6-Phosphatase). In these tissues, irisin’s effects depend on AMPK activation and are partially prevented by compound-C, a non-specific inhibitor of the enzyme [207].

Irisin secretion by skeletal muscle cells was also found to be stimulated in rodent models of physical exercise, including endurance training [208] and voluntary wheel running [209]. Irisin expression in skeletal muscle cells is positively regulated by PGC-1α [206,208]. In order to assess more precisely the in vivo effect of irisin, this hormone was centrally administered in Wistar rats by using osmotic mini pumps. After 7 days, UCP-1 expression was increased in both WAT and BAT, as expected. However, even though energy expenditure was increased due to mitochondrial uncoupling activation, no weight loss was observed, as food consumption was also increased (hyperphagic behavior) [210]. The effect of irisin on the expression of *Ucp1* seems to be tissue-specific, as myokine positively regulates the expression of UCP-1 in human subcutaneous WAT, while perirenal WAT seems to be insensitive to irisin [211]. Others have confirmed that irisin increases UCP-1 abundance in WAT in humans [207]. However, the impact of irisin on WAT browning is still debated in humans. Indeed, a study performed on a large cohort of obese patients exposed to calorie restriction (8 weeks; 289 patients (188 females, 101 males), <800 kcal/day) found no browning effect of subcutaneous WAT [212].

Physical exercise seems to globally stimulate mitochondrial uncoupling leading to the remodeling of skeletal muscle cell physiology. In rats, DNP treatment induced weight loss but also decreased maximal running speed and running economy [213] and ultimately led to contractility failure [214]. Similar effects were noticed in zebrafish [215]. DNP also induces long-term modifications by increasing oxidative fibers and muscular mitochondrial biogenesis in rats [213]. One could argue that these results not only represent the effect of controlled mitochondrial uncoupling on skeletal muscle cell capacity but could also be either the result of off-target effects of DNP or of excessive mitochondrial uncoupling. However, overexpression of *Ucp1* in heart and skeletal muscle cells in mice (to a level comparable to BAT) leads to a decrease in total muscle mass and to a fast-to-slow shift in fiber types. Interestingly, no effect was observed in heart or other muscles [216]. Others reported a considerable decrease in lean mass in these transgenic mice [217].

### 3.7. Adipose Tissue Browning

**Figure 4:**
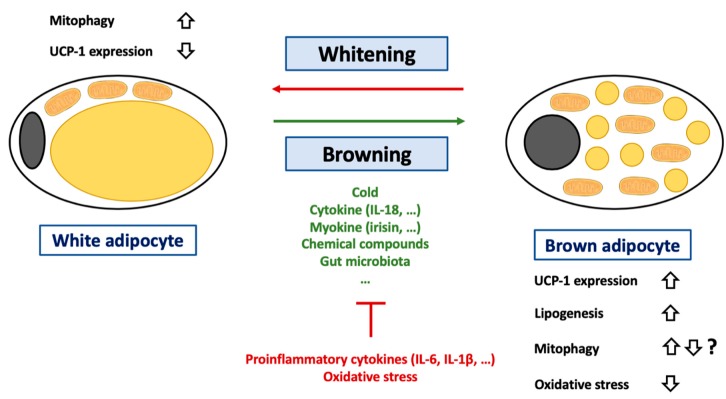
Regulation of browning of white adipocytes. Abbreviations: IL (Interleukin), UCP-1 (Uncoupling Protein-1). White adipocytes are a cell-type specialized in lipid storage but also characterized by an important endocrine function. These cells are characterized by an unilocular lipid droplet and a reduced mitochondrial mass. During the last decades, it was demonstrated that these cells could transdifferentiate into brown adipocytes under certain circumstances such as cold exposure, change in gut microbiota (decrease in *Firmicutes* level) or exposition to cytokine (such as IL-18), myokine (such as irisin) or chemical compounds (such as retinoic acid). This process can be inhibited by several conditions including exposition to proinflammatory cytokines such as IL-6 or IL-1β or to oxidative stress. A more detailed list of pro/anti-browning conditions can be found in the main text. The adipocyte browning is characterized by an increase in lipogenesis and changes in terms of lipid droplet number and composition. The hallmark of adipocyte browning is the induction of UCP-1 expression. Of note, if it is considered that adipocyte browning is accompanied by UCP-1 activation, it was recently suggested that UCP-1 could also play a key role in lipid metabolism. Activation of UCP-1 is often accompanied by a decrease in oxidative stress in adipose tissues. Brown adipocytes can also differentiate into white adipocytes. This process, named adipose tissue whitening, is accompanied an increase in mitophagy in order to clear cells from UCP-1 expressing mitochondria.

Adipose tissues are one of the most abundant tissues in humans, rodents, and many other animals (ranging between 10% to 25% of body weight). Three types of adipose tissues have been identified so far: The WAT [218], the BAT [219,220], and the beige or brite (brown in white) adipose tissue (BIWAT) [220]. WAT is mainly composed of adipocytes, cells specialized in lipid storage and characterized by an unilocular lipid droplet. WAT also acts as an endocrine organ by secreting more than 600 proteins (adipokines) [221]. BAT is essentially composed of brown adipocytes. Opposite of white adipocytes, brown adipocytes display multiple small lipid droplets and a high mitochondrial mass, giving the typical brown color to this tissue. Another hallmark of this tissue is the expression of UCP-1, allowing these cells to oxidize fatty acids in order to dissipate and produce energy as heat, a phenomenon called “non-shivering adaptive thermogenesis.” BIWAT fulfills functions similar to those performed by BAT. Of note, these cell types are possibly not definitive and, according to experimental/physiological conditions, conversion of white adipocytes into brown or brite adipocytes could occur, a process called “browning.” In contrast, brown adipocytes can also turn into white adipocytes if required (BAT whitening). Beige and brown adipocytes are thought to be localized in separated depots [222]. Browning is induced by many physiological or experimental conditions, including cold exposure, physical exercise, or exposure to capsaicin, resveratrol, berberine, quercetin, thiazolidinediones, prostaglandin E2, beta-lapachone, retinoic acid, and cytokines (IL-4, IL-6, (para)thyroid hormone T3, GLP-1, leptin, melatonin, Fgf21, apelin) (Figure 4). All the conditions leading to white adipose tissue browning were thoroughly reviewed recently [222]. 

The hallmark of browning is of course an increase in UCP-1 expression and consequently mitochondrial uncoupling activity (Figure 4). Interestingly, the classic view that mitochondrial uncoupling is triggered by UCP-1 in WAT and BAT has been challenged by recent findings. Indeed, no increase in the mitochondrial uncoupling rate in subcutaneous inguinal WAT of rats exposed to cold was found, even though UCP-1 protein expression was elevated as expected [223]. Moreover, the palmitate oxidation rate was not increased in these cells [223]. Conversely, glycerol kinase, phosphoenolpyruvate carboxykinase levels, as well as glycerol and palmitate incorporation into lipid levels were increased in these cells [223]. Altogether, these data suggest that UCP-1 could mainly control fatty acid export and triacylglycerol in these cells, not just mitochondrial uncoupling [223]. The pertinence of these findings must, however, still be demonstrated in other species and/or other fat depots. 

During browning, UCP-1 must be expressed, imported in mitochondria, and activated; while, conversely, UCP-1 protein must be cleared from the mitochondrial population during whitening (Figure 4). However, the precise mechanisms underlying these switches are not yet fully understood. The beige-to-white adipocyte transition is often associated with a mitochondrial disposal by mitophagy. As proof of the importance of (mito)autophagy in the process, Ucp1 conditional deletion of *Atg5* or *Atg12* is sufficient to prevent the dedifferentiation of beige adipocytes [224]. In addition, mitophagy also seems to be continuously activated in brown adipocytes, possibly in order to degrade dysfunctional mitochondria exposed to prolonged mitochondrial uncoupling (and possible damages induced by oxidative stress) [109]. The importance of mitochondrial uncoupling in mitophagy activation is illustrated by the attenuation of autophagy and mitophagy flows in *Ucp1*^-/-^ murine brown adipocytes [109]. Surprisingly, UCP-1 is not required for cold-induced mitophagy in beige adipocytes [225]. It must also be kept in mind that some authors have indeed demonstrated that mitochondria are progressively degraded by mitophagy and replaced during browning, while others have found that mitophagy was clearly downregulated in the rosiglitazone-induced browning of white 3T3-L1 adipocytes [226], suggesting possible species or cell line specificities.

UCP-1 expression in BAT seems to be regulated by oxidative stress. In line with this theory, the use of antioxidant molecules (butylated hydroxyanisole or N-acetylcysteine) decreases UCP-1 expression (both at mRNA and protein levels) in mouse BAT [227]. Mechanistically, the effect seems to be dependent on Sestrin2. Sestrins are a family of stress-inducible proteins involved in the negative regulation of the AMPK/TORC1 axis [227] and in ROS detoxifying by restoration of the activity of oxidized peroxiredoxins [227,228]. In accordance with its role, Sestrin2 defective mice are characterized by a decrease in BAT UCP-1 expression [227]. Moreover, in the same mouse strain, cold does not upregulate UCP-1 abundance. However, once again, the role of ROS could be dependent on the quantity produced. To illustrate this statement, TLR4 (Toll Like Receptor 4) activation by injection of lipopolysaccharide in C57BL6 mice increases massive ROS production and decreases WAT browning [229].

Oxidative stress is usually observed in adipocytes during obesity. Indeed, during obesity, excessive accumulation of triglycerides in adipocytes leads to cellular and organelle dysfunction [230,231]. In reaction, adipocytes could secrete several adipokines, which will induce a low-grade proinflammatory state. Macrophages can also be recruited to the adipose tissue and further participate in the inflammation state. The interconnection between inflammation, oxidative stress, and adipocyte biology has been extensively reviewed [232,233,234]. In accordance with this theory, many proinflammatory cytokines have been found to be increased in obese patients, including IFN-γ, TNF-α, MCP-1, IL-1β, IL-5, IL-6, IL-10, IL-12, IL-13, and IL-18 [235,236]. In the adipose tissue, the latest cytokine is released by both macrophages and adipocytes in obese mice and humans [237,238]. IL-18 expression seems to be tightly correlated to other proinflammatory markers, such as TNFα, and a linear correlation between IL-18 and BMI was found in humans [237]. IL-18 is known to enhance both T cell and natural killer cell maturation and promote proinflammatory cytokine release [239]. However, its effects are complex and depend on the local environment and on the other cytokines released in parallel. For instance, in combination with IL-2, IL-18 can promote a Th2 response, while a Th1 response will be promoted in response to IL-12 (reviewed here [239]). In addition to its immune role, IL-18 also seems to regulate UCP-1 expression in adipose tissues. Indeed, C57BL7 mice deficient for IL-18 are extremely sensitive to obesity but also exhibit larger brown adipocytes and a higher UCP-1 expression in BAT compared to wild-type littermates [240]. To support the role of IL-18 in this process, exogenous administration of IL-18 in these mice restores the adipocyte size as well as the expression of UCP-1 [240]. The precise action of IL-18 is likely to be complex and possibly independent from the IL-18 receptor (IL-18r). Indeed, mice KO for IL-18r or for IL-18 display different phenotypes. If both mouse models are overweight at the basal state but protected against diet-induced obesity, only IL-18r KO mice can increase thermogenesis and white browning in response to HFD or cold [241]. Such divergences between studies should be explored in the future in order to assess more definitively the role of IL-18 in WAT browning. 

In addition to cytokines, macrophages could also play a direct role in WAT browning. Indeed, activation of the NLRP3 inflammasome in macrophages was recently shown to be sufficient to reduce browning in white human adipocytes differentiated from human adipose-derived stem cells [229]. This effect seems to be due to IL-1β release by macrophages, as IL-1β blocking antibodies protected the human white adipocytes. The effect of IL-1β on white adipocytes includes an increase in ROS production, a decrease in SOD activity, and mitochondrial depolarization [229]. Though most studies often focus on WAT adipokine secretion, BAT is also characterized by its own set of (non-BAT-specific) adipokines, including, for instance, IL-6, IL-8, and MCP-1 [242]. Although IL-6 is usually seen as a proinflammatory cytokine that increases during obesity and is associated with adipocyte dysfunction, the cytokine also regulates BAT differentiation. Indeed, sustained blockage of IL-6 inhibits BAT differentiation in isolated human beige adipocytes [242].

Alterations in gut microbiota were also suggested to be responsible for the low-grade inflammation and cell dysfunctions (including white adipocytes) in obesity (reviewed here [243,244,245]). In addition, gut microbiota also seems to play an important role as a regulator of WAT browning. Indeed, total depletion of gut microbiota via antibiotics or germ-free conditions promotes WAT browning in subcutaneous and perigonadal WAT in mice [246]. The effect is totally counter-balanced by microbial recolonization of the gastrointestinal tract [246]. In some obese insulin-sensitive patients, a decreased level in *Firmicutes* is associated with the downregulation of WAT browning. Interestingly, the *Firmicutes* level was found to be directly correlated with PRDM16 mRNA expression (a key transcription factor involved in BAT differentiation) and UCP-1 expression in subcutaneous and visceral WAT [247]. In accordance with theory, regimen seems to have a direct impact on browning through microbiota regulation. Indeed, intermittent fasting is associated with body weight decrease and WAT browning, also by increasing the *Firmicutes* level [248]. Underlining once again the role of gut microbiota, intermittent fasting does not promote WAT browning in germ-free mice [248]. Similar results were obtained with caloric restriction [249]. Finally, cold exposure also impacts gut microbiota composition in mice, again by increasing the *Firmicutes* level [250].

### 3.8. Cell Signaling

Although the cellular mechanisms triggered by mitochondrial uncoupling are quite well understood, the link between these elements, especially in terms of cell signaling, remains poorly studied. Moreover, it could be difficult to discriminate direct impacts of mitochondrial uncoupling from secondary events associated with cell adaptations.

In terms of cell signaling, the best-known effect of mitochondrial uncouplers is probably a drop in ATP/AMP levels, which induces the activation of the AMPK signaling pathway (Figure 1). This effect has been found in multiple models and species, including white adipocytes [91,251], skeletal muscle cells [252,253], cancer cells [54,254,255,256], and neurons [257]. In addition to AMPK activation (AMPKα1/α2 phosphorylation on Thr172), the mTOR-PI3K-MAPK axis is suppressed in response to mitochondrial uncoupling. Injection of DNP in C57BL/6 mice leads to the suppression of this pathway [94,251] and to the upregulation of genes involved in autophagy, such as LC3B, p62, or Ulk1 [94]. Specific overexpression of UCP-1 in white adipocytes (aP2-UCP-1 mice) also activates AMPK in this cell type. Interestingly, the effects of UCP-1 overexpression are fat depot-dependent and can be explained by differences in term of AMPK subunit activation (reviewed here [258]). Activation of AMPK signaling was also found in mice overexpressing UCP-1 specifically in skeletal muscle cells [252,253]. Akt, a protein positively controlled by AMPK, was also activated in these conditions [252]. Interestingly, even if activated, AMPK phosphorylation does not seem to be required for mitochondrial-induced metabolic effects in skeletal muscle cells. Indeed, mice overexpressing UCP-1 and a dominant negative form of AMPKα2 (UCP1^+/+^ DN-AMPKα2 mice) showed a comparable decrease in body weight and lean and fat mass compared to mice overexpressing UCP-1 [259]. Moreover, these mice also showed a similar intolerance to physical activity and a degeneration of smooth muscle cells [259].

Increased intracellular Ca^2+^ concentration-dependent CREB (cAMP-response element-binding protein) signaling was also found activated in mice injected with DNP [94]. To our knowledge, the activation of this signaling pathway by a mitochondrial uncoupler was only found in that model.

By decreasing ATP levels, mitochondrial uncoupling will also hamper mitochondrial calcium homeostasis. For instance, FCCP leads to an increase in intracellular/cytosolic Ca^2+^ concentration and to the secondary opening of Ca^2+^-activated K^+^ channels, causing a plasma membrane hyperpolarization in mouse sensory neurons [77]. By decreasing the Ca^2+^ buffering capacity of mitochondria, CCCP or FCCP increases global exocytosis in chromaffin cells [164,165,166]. In rat insulinoma cells and primary mouse and rat pancreatic beta cells, FCCP/CCCP inhibits glucose-stimulated (and Ca^2+^-dependent) insulin release (GSIS) [168,169,170].

## 4. Possible Use of Mitochondrial Uncouplers for Human Diseases

As we have seen in this review, mitochondrial uncoupling could induce multiple cellular mechanisms, including autophagy, ROS production/detoxification, cell death, and metabolism. Therefore, under particular circumstances and intensity, mitochondrial uncoupling can have beneficial effects. In 1934, and despite existing reports about the toxicity of high doses of DNP, the drug was rapidly and routinely adopted for obesity treatment in the USA. A couple of years later, a report of DNP-imputable deaths (all associated with excessive ingestion of DNP) led to the ban of the mitochondrial uncoupler from medical use. Moreover, interindividual sensitivity largely differs, i.e., side effects could be observed in some patients but not in others, even at therapeutically efficient doses, thereby complicating its medical use. The interindividual sensitivity also varies according to time [260]. The reported side effects of DNP include acute cataract [261,262], sensory axonal polyneuropathy [263], fever, tachycardia, sweating, nausea, rash, breathing difficulties, abdominal pain, agitation, headache, and death [264,265]. More recently, an increase in the use of DNP as a self-medication has been observed in China [266] and the United Kingdom [267], leading to new cases of DNP-associated fatalities. Taking into consideration its side effects, DNP, as well as any other non-specific mitochondrial uncouplers, should not be used in humans, even under tightly controlled conditions. However, recent findings still indicate that the induction of mitochondrial uncoupling could be beneficial in some human diseases. Pending development of mitochondria-specific and safe uncoupler agents (meaning they are able to be delivered to specific cell types and allow fine-tuning control of the level of uncoupling) suitable for human use could lead to the efficient treatment of these diseases. A better understanding of molecular mechanisms underlying these effects would also be highly valuable. In the following paragraphs, and even if self-medication using current mitochondrial uncouplers should never be done, we will summarize some recent findings highlighting the possible effect of mitochondrial uncoupling in some human diseases.

**Figure 5:**
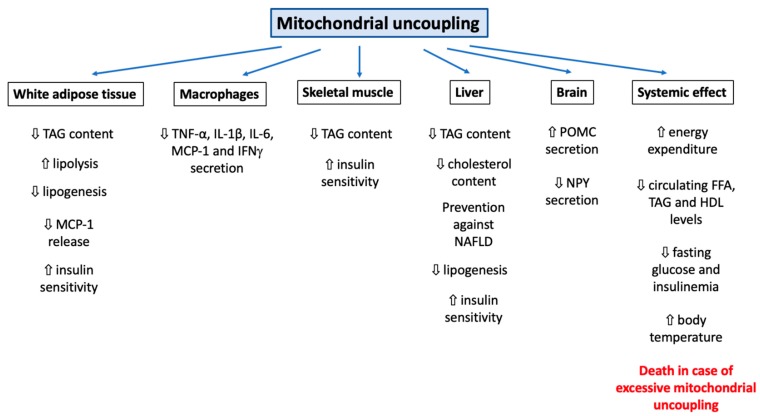
Systemic effect of mitochondrial uncouplers on different organs in the context of obesity and type 2 diabetes. Abbreviations: HDL (High Density Lipoprotein), IFNγ (Interferon γ), IL (Interleukin), MCP-1 (Monocyte Chemoattractant Protein-1), NAFLD (Non-Alcoholic Fatty Liver Disease), NPY (Neuropeptide Y), POMC (Proopiomelanocortin), TAG (Triacylglycerol), TNF-α (Tumor Necrosis Factor-α). Use of systemic mitochondrial uncouplers, such as DNP or FCCP, will impact the functions of multiples organs. Some of these effects could be beneficial for the treatment of obesity and associated complications, such as type 2 diabetes. First, the global mitochondrial uncoupling induced by these molecules and the subsequent increase in cell respiration will increase global energy expenditure. As a consequence, lipid stores of different organs will be mobilized (increase in lipolysis, decrease in lipogenesis, and decrease in total TAG content and circulating levels). A global decrease in lipid stores will also help to alleviate the proinflammatory state seen in adipose tissues of obese patients. Patients will also be protected against NAFLD. Mitochondrial uncouplers also exert a direct effect on the brain and could affect the activity of NPY (decrease) and POMC (increase) neuron activity. Finally, it is important to stress that excessive mitochondrial uncoupling (due to excessive dosage and/or sensitivity to these compounds) will induce many complications, such as acute cataract, sensory axonal polyneuropathy, fever, tachycardia, sweating, nausea, rash, breathing difficulties, abdominal pain, agitation, headache, and, ultimately, death. Therefore, if mitochondrial uncoupling induction could be beneficial, it should be achieved by using highly mitochondria-specific and safe compounds, which remain to be developed.

As suggested by the very first use of DNP in humans, obesity and its complications can also be limited by using uncoupling agents [268]. The effects of mitochondrial uncouplers in the context of obesity and type 2 diabetes are depicted at Figure 5. The triggered mechanisms are not yet totally clear. For instance, DNP’s effect on obesity seems to be dependent on environmental temperature, a well-known phenomenon in mice. Indeed, DNP induces a decrease in energy expenditure by 17% and weight loss by 23% at 30 °C in female C57BL/6J, while no significant effect has been observed at 22 °C [269]. Interestingly, food consumption of mice housed at 22 °C was higher than at 30 °C, which could counter-balance the effect of DNP [269]. Although mitochondrial uncouplers are often seen as molecules that increase energy consumption, they could also have other effects that could account for their anti-obesity action. For instance, DNP can affect melanocortin-secreting neuron activity by inhibiting orexigenic NPY (Neuropeptide Y) and activating anorexigenic POMC (Proopiomelanocortin) neurons [270]. The DNP effect requires, at least partially, the melanocortin-4 receptor (MC4R) as observed in C57BL/6 MC4R KO mice [270]. Proinflammatory adipokine release (TNF-α, IL-1β, IL-6, MCP-1, and IFNγ) also seems to be decreased by high DNP concentrations (100 μM) in isolated murine RAW 264.7 macrophages [271]. MCP-1 release was also decreased by lower concentrations (1.5 μM) of DNP in murine 3T3-L1 adipocytes, an effect which relies on endoplasmic reticulum stress induction and AMPK activation [272].

Type 2 diabetes is a well-known possible complication of long-term obesity. By acting on white adipocyte mass, mitochondrial uncouplers could protect against diabetes, as limiting hyperaccumulation of triacyglycerol (TAG) in these cells might limit organelle stress [91,273,274,275] and modifications in the expression pattern of genes encoding adipokines, which are known to communicate the level of adiposity to other organs [221,276]. Rats exposed to 1 mg/kg DNP using an orally available, controlled-release formulation of DNP exhibit a decrease in fasting plasma glucose, fatty acids (by 30%), TAG concentrations, HDL concentrations (by 30%) and hyperinsulinemia (50%) [277]. Global glucose tolerance was also improved in DNP-treated rats, an effect resulting from an increase in liver- and muscle-insulin sensitivity [277]. DNP treatment also prevents NAFLD (Non-Alcoholic Fatty Liver Disease) development in rats by reducing fasting plasma glucose as well as concentrations of non-esterified fatty acids and insulin, accompanied by a 50–90% decrease in TAG concentrations in plasma, liver, and skeletal muscle [277]. Similar effects of this treatment have also been reported by the same group in lipodystrophic mice (AZIP/F-1), a mouse model expressing the A-ZIP/F gene under the control of the Fabp4 promoter characterized by a virtually complete absence of white adipose tissue [278]. As mentioned before, the delivery of an inadequate dose of mitochondrial uncoupler could be a challenge and could limit the safety of these drugs. To cope with this problem, a research group reported the development of an injectable crystal gel of DNP (DNP-LC-gel) allowing low and sustained concentrations of the uncoupler. When used in rats, the treatment reduces hepatic steatosis development and lowers TAG liver levels as well as total cholesterol content, while body temperature is not increased [279]. Interestingly, mitochondrial uncoupling could also be achieved without using synthetic uncouplers. Indeed, salsalate, a prodrug of salicylate, was shown to lower blood glucose in type 2 diabetic patients [280]. The mechanism seems to depend on (1) a direct activation of AMPK via the β1 subunit by salsalate and (2) an AMPK-independent increase in mitochondrial proton conductance [281].

As DNP derivatives or other non-specific mitochondrial uncouplers cannot be used safely in humans, an alternative approach could be to stimulate the activity of endogenous mitochondrial uncoupling/thermogenic capacity by using synthetic, natural compounds or just by changing food composition (diet-induced thermogenesis). For instance, a long-chain PUFA (PolyUnsaturated Fatty Acids)-enriched HFD protects C57BL/6J mice against obesity, while an isocaloric diet strongly induces weight gain in the same mouse model [282]. Molecular mechanisms include diminished de novo lipogenesis and increased hepatic and intestinal fatty acid oxidation [282]. An increase in UCP-1 protein expression and activity (acting as an efficient monocarboxylic FA anion flippase and regulated by GDP [283]) accounted for the promoted thermogenic capacity [282]. Mechanistically, the treatment seems to induce AMPKα phosphorylation (possibly due to UCP-1 uncoupling/thermogenic activity and a decrease in ATP/AMP level), PPARα activation, and the expression of Fgf21 [282]. The direct effect of Fgf21 on adipocyte browning has also been demonstrated by others [284]. Other reports suggest that PUFA supplementation indeed protects against diet-induced obesity by decreasing the expression and/or activity of SREBP-1c and PPARα [285]. In addition, the administration of other PUFAs, such as eicosapentaenoic (EPA) and docosahexaenoic (DHA) to C57BL/6J mice upregulates the expression of mitochondrial protein markers, such as CPT-1A (carnitine palmitoyl transferase-1A), and regulators of the biogenesis of the organelle, including PGC1α and Nrf1, mainly in epididymal white adipocytes [286]. Whole genome microarray analysis performed on intestinal cells of C57BL/6J mice treated with EPA and DHA confirmed that the main biological process affected by such treatment is lipid metabolism (increase in the expression of *Acaa1, Acacb, CPT-1A*) [287]. Cholesterol synthesis was also reduced, while its reabsorption was increased [287]. Importantly, we must note that some anti-obesity effects of PUFAs could be independent of UCP-1-dependent mitochondrial uncoupling. Indeed, although HFD supplemented with n-3 PUFA protects C57BL/6J mice against weight gain, no effect on the expression and/or activation of the gene encoding the thermogenic UCP-1 could be found [286].

However, mitochondrial uncoupling is not only related to metabolism, obesity, diabetes, or Alzheimer’s disease. Indeed, a putative role in stroke, dementia, and depression has been suggested. Indeed, mitochondrial dysfunction, a common hallmark in neurodegenerative diseases, points especially to the mitochondrial uncoupling process as a critical player [288]. These authors identified an intronic variant of the neuronal UCP4 (*UCP4/SLC25A27*) gene that affects the risk of late-onset Alzheimer’s disease (LOAD) and late-onset familial and sporadic cases of frontotemporal dementia [289]. Mutations in VCP (Valosin-Containing Protein), a type II AAA+ ATPase family, could also cause mitochondrial uncoupling and play a role in frontotemporal dementia (IBMPFD) and some familial cases of amyotrophic lateral sclerosis, as it could sensitize deficient neuronal cells to subsequent stress, such as ischemia and other conditions requiring high energy demand [290].

A clear role of UCP2 in the reduction of ROS generation allowing protection against reperfusion damage comes from a study showing that -866G/A polymorphism in the promoter of the UCP2 gene (enhancing its transcription) is associated with functional prognosis in patients with embolic ischemic stroke after early recanalization [291]. This study clearly demonstrates that, in humans, increased expression of UCP2 is neuroprotective [291]. This beneficial effect of UCP2 was also observed in traumatic brain injury [292] or neurodegenerative conditions [293].

## 5. Conclusions

Mitochondrial uncoupling is often perceived as an isolated cellular mechanism or a dysfunction. However, the induction of such a condition not only impacts mitochondrial respiration but can also activate or hamper multiple cellular mechanisms, including bulk and specific forms of autophagy, ROS production regulation, protein secretion, physical exercise capacity, and adipose tissue biology (Figure 1). These responses are complex and often rely on the combination of several biological features (species, cell type), physical (temperature), and/or chemical (ion concentration, lipid composition of membranes, nature of the uncoupler) parameters. Unfortunately, the cellular consequences have not been studied rigorously. Indeed, most mechanistic studies have focused on the impact of classical but non-specific mitochondrial uncouplers, such as DNP or FCCP. Therefore, it could be difficult to discriminate effects directly imputable to mitochondrial uncoupling from unspecific effects. The continuous development of new mitochondria-specific uncoupling agents should greatly improve our knowledge of the cellular consequences of mitochondrial uncoupling. Moreover, the identification of such compounds, safer than classic DNP, should also eliminate their use in the treatment of human diseases, such as obesity (and associated complications) or some forms of cancers, in order to improve safety.

## Figures and Tables

**Table 1:** List of mitochondrial uncouplers.

Uncoupling Agent	Abbreviation (If Any)	Mitochondria Specific	Mechanism	Reference
(2-fluorophenyl)6-[(2-fluorophenyl)amino](1,2,5-oxadiazolo [3,4-e]pyrazin-5-yl)amine	BAM15	Yes	Protonophore	Kenwood et al. [45]
(E)-4-(1 H-indol-3-ylvinyl)-N-methylpyridinium iodide	F16	Yes	Lipophilic cation	Wang et al. [46]
1,3-bis(3,5-dichlorophenyl)urea	CR4	No	Protonophore	Figarola et al. [47]
Adenine Nucleotide Translocase 1	ANT-1	Yes	ATP/ADP exchange and FFA transporter	Andreyev et al. [23]
Bupivacain	-	No	Local anesthetic with protonophoric activity (at least partially)	Sztark et al. [48]
C12TPP	Dodecyltriphenylphosphonium	Yes	Protonophore	Severin et al. [49]
Carbonyl cyanide p-trifluoro-methoxyphenyl hydrazone	FCCP	No	Protonophore	Benz et al. [50]
Carbonylcyanide-3-chlorophenylhydrazone	CCCP	No	Protonophore	Kasianowicz et al. [51]
CO-releasing molecules	CORM	No	Activates mitochondrial large-conductance calcium-regulated potassium ion channels	Kaczara et al. [52]
Dinitrophenol	DNP	No	Protonophore	Loomis et al. [53]
FR58P1	-	Yes	Protonophore	Urra et al. [54]
Free fatty acids	FFA	No	Protonophoric action and activation of UCP-1 activity	Wojtczak et al. [29], Divakaruni et al. [32]
Halothane	-	No	Anesthetic, partial, protonophore and inhibits mitochondrial complex I	Hanley et al. [55]
Inactin	-	No	Thiobarbiturate with protonophoric activity (at least partially)	Schiffer et al. [56]
Isoflurane	-	No	Anesthetic, partial, protonophore and inhibits mitochondrial complex I	Hanley et al. [55]
Mitofluo	-	Yes	Protonophore (fluorescent)	Denisov et al. [57]
MitoPhotoDNP	MitoPhotoDinitrophenol	Yes	Protonophore (photoactivable)	Chalmers et al. [58]
N-acyl amino acids	-	No	Protonophore	Lin et al. [59]
Rhodamine 19 butyl ester	C4R1		Protonophore, mild uncoupler	Khailova et al. [60]
Sevoflurane	-	No	Anesthetic, partial, protonophore inhibits mitochondrial complex I	Hanley et al. [55]
Thyroid hormone T3	T3	No	Regulates mitochondrial uncoupling by different mechanisms: (1) by sympathetic stimulation, (2) by increasing acylcarnitine production, thereby activating mitochondrial respiration/uncoupling, and (3) by directly stimulating the transcription of *Ucp1* gene	Yau et al. [61]
Uncoupling Protein-1	UCP-1	Yes	Transmembrane protein channel present at the inner mitochondrial membrane catalyzing the transport of protons across the mitochondrial membrane and thereby inducing mitochondrial uncoupling	Jacobsson et al. [7]

This table lists the main mitochondrial uncouplers referenced in the review, including their full names, their specificity, and their mode of action.

## Supplementary Materials

Supplementary File 1
