# Supplementary material for: Mitochondrial Uncoupling: A Key Controller of Biological Processes in Physiology and Diseases
Source: Cells. 2019 Jul 30;8(8):795. doi: 10.3390/cells8080795 (PMC6721602; doi:10.3390/cells8080795)
Supplement: Supplementary File 1 [file cells-08-00795-s001.zip › Rebuttal letter.docx]

To the Reviewers,

In this document, the four reviewers will find the answers to their criticisms, comments, remarks and suggestions for improvement of the manuscript and the way we addressed them in the revised version. We thank all of them for the accurate and fair comments for helping us to deliver a more focused and, for some sections, a more nuanced version of the review.

**Reviewer 1 :**

**The main criticism of Reviewer 1 is that the authors refer to a large range of areas and thus, there is a lack of focus making the reading difficult to follow. In addition, some critical parts should be carefully discussed.**

**Remark 1. Authors should carefully discuss regarding human use of mitochondrial uncoupling agents of DNP, because it is well known even nowadays, not only to be reported in US in 1940s, to produce a death after use (J Chin Med Assoc. 2014 Aug;77(8):443-5). Based on essential moral of scientists, I would like to rethink the content of the texts. At least, authors should not encourage the medical use such a dangerous chemical.  Indeed, some investigators are noticing the potential benefits of mito-uncoupling agents to medical use and tried in various animal model. However, in case of DNP, people must not use even in the individualized and limited condition. Because DNP can be obtained easily with low-price by poor understanding people, it is likely to increase the health damage. If authors eager to contribute to achievement of human health, they should first encourage to develop a new and safe mito-uncoupling agent, which is not DNP.**

We totally agree with the reviewer’s comment and it was certainly not our intention to encourage the use of DNP as a therapeutic agent. It is true that as long as a mitochondrial uncoupler or a modulator of physiological uncoupling mechanism that will allow specific cell/tissue targeting and control of (mild)uncoupling intensity is not developed, this systemic and uncontrolled level of mitochondrial uncoupling CAN NOT be used as a broad approach and certainly not without a strict medical control and guidance. To take into account the recommendations of the reviewer, the following modifications have been made to the document:

1) We reduced the size and length of the paragraph related to DNP, especially because J.G. Geisler published a full paper on the molecule as a medicine (Cells, 2019). We now referred to this paper and mention clearly the restriction to use DNP as a drug.
2) To address the criticism of the reviewer, we also added some info to the existing paragraph on DNP with a clear indication that it **cannot be used** for humans.
3) We also included the reference mentioned by the reviewer as well as an another one describing a similar dramatic problem related to cell death after use observed in the UK.
4) Eventually, we also emphasized the danger of using DNP in the new Figure 2 of the revised version.

**Remark 2. In line 825-826, authors should provide correct references. Some of them do not conclude what they refer.**

This is true that these references were misplaced, and we now removed these sentences containing these references as they were not essential or critical for the message to deliver. This helped to shorten the review.

**Remark 3. Please try to shorten whole sentence, which is too long and unorganized.**

The manuscript has been revised accordingly and English has now been completely revised by native English speakers and professional scientists.

**Reviewer 2**

**Remark 1: Reviewer 2 complimented the authors for the extent of interesting information in the review even if he found the review confusing in the context of uncouplers/leak/pump slip. Some of that confusion is due to the poor English syntax.**

We agree with the reviewer and the manuscript has now been completely revised by native English speakers and professional scientists.

**Remark 2: other elements of the confusion stem from a lack of scientific explanation, in places, on the role of uncouplers/proton leak/pump slip.**

To relieve the scientific confusion, we have now added some sentences about the different sources of proton leak in the introduction and clear description of what should be understood by uncoupler, proton leak and pump slip and refer to the review by Hinkle (BBA 1706, 1-11, 2005) for more details.

**Remark 3: I am also a bit confused about the ultimate aim of the review.  Is the goal to convey that uncouplers/slip inducers are potentially new medicines (again)? Is the goal to explain that slip & leak have an important physiological relevance?**

Yes, the main goal of the review is to explain and describe, in a summarized version, how slip and leak have patho-physio-logical relevance. While the use as medicine is still conserved, this part as been reduced and we now emphasize clearly the fact that while interesting studies have been done on animals, its human use should be very restricted and only performed under medical control. The need for development of more specific targeting to particular cell and tissues and control of uncoupling level is also emphasized.

**Remark 4: there is also a certain lack of organized delineation of information throughout. It needs a few more iterations with the authors and may their colleagues and some emphasis on the English syntax.**

As mentioned, the revised version of the manuscript has been completely revised by native English speakers and professional scientists.

**Remark 5: for instance, from a scientific point of view, I would argue that there are three kinds of mitochondrial proton leak (a) natural/basal leak, (b) UCP catalyzed leak and (c) chemically induced leak. Natural/basal is hardly discussed. UCP catalyzed leak is FA dependent, but little is mentioned of purine inhibition of UCP's nor how BAT cells are activated by noradrenaline.**

We have now added some sentences about the different sources of proton leak in the introduction. We have also added information related to the regulation of UCP activity as well as signaling triggered by noradrenaline controlling BAT cell activation.

**Remark 6: Brownlee, for instance, has written about mitochondrial ROS as the link to all diabetic complications. He has used ectopically expressed UCP1 and uncouplers to provide evidence for that. It is also not very clear, form the literature, whether UCP2/3 are 'pure' uncouplers. It might be that their transporter activity may result in 'uncoupling' (Vogel et al). There is no mention of that in the review.**

We have now added a full paragraph about the different UCPs and their proposed mechanisms of action. In addition, a link to a complete review about the topic has now been included to invite the reader to more details about the important point raised by the reviewer. The link to the section about the UCPs has been added when we discuss the role of lipophilic weak acids in the revised version of the manuscript. Some pertinent references to the work of Brownlee have been added to the manuscript.

**Reviewer 3**

**The Reviewer mentioned that it is a comprehensive literature review of the mechanisms of mitochondrial uncoupling and associated biological effects/consequences. The reviewer found that it would be a valuable resource in the field and that the topic and content are well worthy-of publication even in its present form. He raised several criticisms/remarks or suggestions that we took in to account.**

**Remark 1: this review is generally well-written, however, there are numerous minor grammatical errors scattered throughout the manuscript, and therefore, careful English usage editing should be performed prior to publication.**

The revised version of the manuscript has been completely revised and edited by native English speakers and professional scientists.

**Remark 2: the first paragraph of Section 2.2 indicates that UCP-1 and lipophilic weak acids acting as protonophores have completely different molecular mechanisms. A clear description of each mechanism would be very beneficial.**

According to the suggestion, we have now added a full paragraph about UCPs and their mechanisms of action. In addition, a link to a complete review about the topic has been included in the revised version of the manuscript to invite the reader to more detailed information about this topic. This information has been introduced in the section about the UCPs when the role of lipophilic weak acids is discussed.

**Remark 3: this same paragraph indicates that the lipophilic weak acids acting as protonophores catalyze the transport of protons through the cell membranes. The authors should verify that "catalyze" is the appropriate term here.**

It is true that it is not an appropriate term as it does not refer to an enzymatic activity.

“The new sentences are thus: *“These molecules are lipophilic weak acids that act as protonophores. Due to their hydrophobic nature, these compounds can easily traffic across biological membranes and allow the protons to cross these membranes, mimicking the effect of UCP-1. However, the molecular mechanism is completely different (see Section 1: UCPs and ANTs).”*

**Remark 4: throughout section 2 (and some later sections), the authors often refer to the effects of uncouplers on respiratory complexes. These descriptions are necessary, but it is often difficult to understand whether the authors also consider uncoupler effects on respiratory complexes to be an uncoupling effect (which is incorrect). The authors should consider clarifying these statements to discriminate between true uncoupling and inhibitory effects on respiratory complexes.**

It is true that these mixed statements are confusing. This distinction has now been clearly made throughout the text and in Table 1.

**Remark 5: the authors indicate multiple times that UCP2 and UCP3 are not "true" uncouplers. While these statements may be accurate, the authors should explain these statements. Explain to the readers why UCP1 is a true uncoupler but UCP2 and UCP3 are not.**

We have now added several sentences to explain the differences between UCP1 (a true uncoupler) and other UCPs such as UCP2 and UCP3 that are not perceived as “true” uncouplers.

Text has been revised and extended accordingly by adding the following paragraph.

*“Importantly, although strong evidence of the mitochondrial uncoupling property is clear for UCP-1 [7,12], it is still heavily debated for the other UCPs [13-15]. Regarding UCP-2, Bouillaud and collaborators first suggested that this protein could have uncoupling properties in yeast expression systems [16], but they later reported that it does not possess any uncoupling effect in mice [17]. However, others found that UCP-2 could indeed induce mitochondrial uncoupling by essentially acting as a fatty acid flippase transporter [18]. The same authors suggested that UCP-2 activity could by completely blocked by GDP at the basal state (100 µM, which corresponds to the basal intracellular GDP/GTP concentration) [18]. UCP-2 could then only be activated under some circumstances, such as a massive entry of FFAs into mitochondria [18]. Regarding UCP-3, several groups reported that UCP-3 has no clear uncoupling effect as it does, for instance, in CHO cells overexpressing this protein [19] or in rat skeletal muscles [20]. However, as for UCP-2, UCP-3 uncoupling activity could only be activated under particular circumstances, such as high availability in FFAs. Importantly, one must keep in mind the endogenous level of expression of each UCP. For instance, UCP-3 expression is expressed at a much lower level than UCP-1 (200–700 fold) in mouse skeletal muscle [21]. Therefore, its function (as well as any other UCP) should be studied in models with similar expression levels. The mitochondrial uncoupling effect observed for these UCPs in an overexpression system could otherwise be likely artefactual. Finally, other functions have been suggested for UCP-2 and UCP-3 and include redox regulation or glucose sensing [13,22].”*

**Remark 6: the authors indicate that they do not want to explain the role of UCP2 and UCP3 on ROS production in section 3.3 because other reviews do this. Still, the authors could briefly summarize in one or two sentences the effects of UCP2 and UCP3 on ROS.**

The sentence below about the effects of UCP-2 and -3 on ROS production has now been introduced in the revised version of the manuscript.

*“The role of other UCP members (especially UCP-2 and -3) on ROS production is more documented and was recently reviewed [148]. These UCP members are known to alleviate oxidative stress by decreasing mitochondrial ROS production [148]. The overexpression of these UCPs also protects against excessive superoxide production and oxidative damage observed in pathological situations, such as stroke and ischemia/reperfusion [148].”*

**Reviewer 4**

Reviewer 4 mentioned that it is an important paper that describes the role of MU in biology of diseases. To his knowledge, this is the first paper summarizing clinical importance of MU and the uniqueness of paper as well as relevance to the field of biology and medicine have a significant merit. The role of exercise and gut microflora are extremely important to cover, and the authors did a good job at that.

**Remark 1: the Reviewer also think that it would be worthwhile to mention MU in heart disease and psychiatric disease such as depression by adding just a couple of paragraphs when we talk about diabetes and Alzheimer's.**

As several reviewers and editors asked to cut and shorten the manuscript, especially in the part related to mitochondrial uncouplers and diseases, we decided to develop few sentences and short paragraphs on stroke, dementia and other neurologic troubles. This is justified to give the reader that mitochondrial uncoupling might be important in other diseases as well than just those mentioned and related to metabolism. However, we will refer to extensive reviews and papers on those satellite topics. The role of MU in heart diseases would require a review fully devoted to the topic due to the number of studies referring to mitochondrial uncoupling in cardiovascular diseases.

**Remark 2: the Reviewer also requested that we cite the works of Mark Tarnopolsky who has done and a lot of research on mitochondria and exercise.**

We now quote several papers from Tarnopolsky’s research group including their recent review (Nilsson and Tarnopolsky, *Biology* **2019**, *8*(2), 40) who did excellent work on the link between exercise and mitochondrial uncoupling.

**Remark 3: eventually, the reviewer mentioned that the manuscript would benefit of the addition of another Figure containing a schematic diagram of MU mechanisms in either obesity/diabetes or exercise these aspects are extensively covered in the review.**

As suggested by the reviewer and as we found that it was a good idea to support the long description of systemic effects of mitochondrial uncouplers in the context of obesity and type 2 diabetes, we have prepared and included one extra Figure (Figure 2) supporting this section.
